# Landslide Susceptibility Evaluation Based on Potential Disaster Identification and Ensemble Learning

**DOI:** 10.3390/ijerph192114241

**Published:** 2022-10-31

**Authors:** Xianmin Wang, Xinlong Zhang, Jia Bi, Xudong Zhang, Shiqiang Deng, Zhiwei Liu, Lizhe Wang, Haixiang Guo

**Affiliations:** 1 Hubei Subsurface Multi-Scale Imaging Key Laboratory, School of Geophysics and Geomatics, China University of Geosciences, Wuhan 430074, China; 2 Key Laboratory of Geological and Evaluation of Ministry of Education, China University of Geosciences, Wuhan 430074, China; 3 Institute of Geological Survey of Tibet Autonomous Region, Lhasa 850000, China; 4 The Fifth Geological Brigade, Bureau of Geology and Mineral Exploration and Development of Tibet Autonomous Region, Glomud 816000, China; 5 Laboratory of Natural Disaster Risk Prevention and Emergency Management, School of Economics and Management, China University of Geosciences, Wuhan 430074, China

**Keywords:** landslide susceptibility evaluation, hidden landslide, remote sensing, machine learning

## Abstract

Catastrophic landslides have much more frequently occurred worldwide due to increasing extreme rainfall events and intensified human engineering activity. Landslide susceptibility evaluation (LSE) is a vital and effective technique for the prevention and control of disastrous landslides. Moreover, about 80% of disastrous landslides had not been discovered ahead and significantly impeded social and economic sustainability development. However, the present studies on LSE mainly focus on the known landslides, neglect the great threat posed by the potential landslides, and thus to some degree constrain the precision and rationality of LSE maps. Moreover, at present, potential landslides are generally identified by the characteristics of surface deformation, terrain, and/or geomorphology. The essential disaster-inducing mechanism is neglected, which has caused relatively low accuracies and relatively high false alarms. Therefore, this work suggests new synthetic criteria of potential landslide identification. The criteria involve surface deformation, disaster-controlling features, and disaster-triggering characteristics and improve the recognition accuracy and lower the false alarm. Furthermore, this work combines the known landslides and discovered potential landslides to improve the precision and rationality of LSE. This work selects Chaya County, a representative region significantly threatened by landslides, as the study area and employs multisource data (geological, topographical, geographical, hydrological, meteorological, seismic, and remote sensing data) to identify potential landslides and realize LSE based on the time-series InSAR technique and XGBoost algorithm. The LSE precision indices of AUC, Accuracy, TPR, F1-score, and Kappa coefficient reach 0.996, 97.98%, 98.77%, 0.98, and 0.96, respectively, and 16 potential landslides are newly discovered. Moreover, the development characteristics of potential landslides and the cause of high landslide susceptibility are illuminated. The proposed synthetic criteria of potential landslide identification and the LSE idea of combining known and potential landslides can be utilized to other disaster-serious regions in the world.

## 1. Introduction

Accompanied by global climate change, extreme rainfall increase, urban expansion, and human engineering activity intensification, catastrophic landslides have much more frequently occurred around the world, caused enormous losses to human lives and properties, and seriously hindered the sustainable development of nations and society. Thus, an imperious demand is suggested for the valid prevention and control of landslides to effectively mitigate the massive damage to people and engineering infrastructures. Landslide susceptibility evaluation (LSE) can predict the dangerous region where landslides may occur [1]; thus, it has become a crucial technique for disaster prevention and attracted great attention from the worldwide scientists [2,3,4,5,6,7]. Moreover, about 80% of these disastrous landslides have not been discovered in advance [8], called as potential or hidden landslides, which causes disaster control measures to miss the best time. Therefore, the early identification of potential landslides and accurate evaluation of landslide susceptibility play an essential role in control and mitigation of landslide hazards. This work suggests new synthetic criteria of potential (active) landslide identification to improve the accuracy of landslide recognition. Also, this work conducts LSE by integrating both the potential and known landslides to enhance the precision and rationality of LSE.

Potential landslides are generally situated on steep mountains and are difficult to arrive at, discover and investigate via manpower. Interferometric Synthetic Aperture Radar (InSAR) technique has become a powerful means to discover potential landslides due to the advantages of accurate detection of surface deformation and simultaneous observation in a wide, precipitous area [9,10,11,12,13,14]. Thus, some studies employed surface deformation extracted by Differential InSAR (D-InSAR) (e.g., [15]), Small Baseline InSAR (SBAS-InSAR) (e.g., [16,17]), Persistent Scatterer InSAR (PS-InSAR) (e.g., [9,18]), or hybrid time-series InSAR techniques (e.g., [19]) to recognize potential landslides. InSAR productions feature strong noises, and many factors, except active landslides, can lead to ground deformation, for example, foundation settlement, mining activity, groundwater exploitation, foundation pit excavation, and crustal movement. Thus, some studies combined the surface deformation observed by InSAR with the geological, topographical, and/or geomorphological features to identify potential landslides (e.g., [18,20,21,22]). These above works have made important breakthroughs in hidden landslide discovery.

There have been a large number of excellent studies in LSE, and the LSE methods in these studies generally fall in two categories: knowledge-driven methods and data-driven methods [23,24]. The knowledge-driven LSE methods mainly include analytic hierarchy process (AHP) [5,25,26], fuzzy-AHP [27], fuzzy-relation AHP [28], fuzzy logic [29,30], fuzzy comprehensive evaluation [31], and fuzzy unordered rule induction [32] methods. The data-driven methods primarily consist of logistic regression [33], frequency ratio [34], weights of evidence [35], Information value [36], shallow machine learning (e.g., support vector machine [37], artificial neural network [38], random forest [39], and decision tree [40]), and deep learning (e.g., convolutional neural network [41], deep neural network [42], recurrent neural network [43], and deep belief network [44]) methods. Moreover, some ensemble methods were employed in LSE, including the combination of ant colony optimization and deep belief network [45], the ensemble of a radial basis function neural network, random subspace, attribute selected classifier, cascade generalization, and dagging [46], bagging based reduced error pruning trees [47] and so on. However, the vast majority of present studies conducted LSE based on optical images and known landslides and neglected the serious threat posed by potential landslides. In recent years, a few studies became to involve InSAR technique into LSE and mainly include the following four aspects. (1) The surface deformation characteristics extracted by InSAR technique were employed to validate the LSE results acquired from optical images and known landslides [48,49]. (2) LSEs were conducted by combining InSAR-detected surface deformation and other influencing factors. The factors of surface deformation, lithology, topography, land use and so on were used together as the input parameters to evaluate landslide susceptibility [50,51,52]. (3) The surface deformation features detected by InSAR technique were adopted to improve the LSE results obtained from optical images and known landslides [53,54,55] or to refine the LSE map acquired from a physical model [56]. (4) Landslide susceptibility was assessed according to the potential landslides instead of the historical landslides [57,58]. The aspects (1) and (2) actually conducted LSE according to known landslides or active known landslides. The aspect (3) virtually employed surface deformation derived by InSAR technique to recognize potential landslides. To our knowledge, only one study carried out LSE using both potential and historical landslides. Kontoes et al. [59] employed the characteristics of ground deformation detected by multi-temporal InSAR interferometry technique, geomorphology, slope angle, and land cover (vegetation deterioration) to determine the active landslides from 1992 to 2010. Both the past landslides and the identified active landslides constituted the landslide inventory, and the method of weights of evidence (WoE) was used to generate the LSE map. These studies on LSE have made significant contributions to improve the accuracy.

Despite great progress achieved in early identification of potential landslides and in assessment of landslide susceptibility, there are still two problems restricting the accuracy and rationality. (1) The present studies generally employed surface deformation or the combination of surface deformation, geomorphological, and topographical features to recognize potential landslides. The essential disaster-triggering mechanism has been neglected, which leads to the relatively low identification accuracy and relatively high false alarm and may cause a huge waste of time, money, and manpower on field investigation to validate the fake landslides. The Ministry of Natural Resources, PRC indicated that the precisions of potential landslide recognition via InSAR, optical image, and expert interpretation techniques are 69.9%, 62.8%, 44%, and 29.8%, respectively in Sichuan Province, Hubei Province, Shanxi Province, and Chongqing City that are vulnerable to landslides. This is because the landslide-influencing factors contain not only the controlling factors, such as topography, geology, and geomorphology but also the triggering factors, including earthquakes, precipitation, and human engineering activity. Landslides occur under the joint effect of the controlling and inducing factors. Thus, the synthetic criteria integrating slope deformation, disaster-controlling mechanism, and disaster-inducing mechanism are necessary to improve the accuracy of potential landslide recognition. (2) The vast majority of present studies adopted optical images and historical landslides to perform LSE, omitted the development and threat of potential landslides, and to a certain degree limited the precision, rationality, and practicability of LSE maps. The historical and potential landslides both reflect landslide activity and susceptibility. Therefore, a complete landslide inventory, including the two types of landslides, can improve the accuracy, rationality, and practicability of LSE maps. Focusing on the above two problems, this work makes two improvements. (1) Synthetic criteria are established to improve the accuracy of potential landslide identification, and the criteria are composed of the characteristics of ground deformation, geology, topography, geomorphology, environment, earthquake, rainfall, and human engineering activity. (2) Landslide susceptibility is assessed according to both the potential and historical landslides to improve the precision and rationality of a LSE map.

This work adopts the multisource data including geological, topographical, geographical, hydrological, meteorological, seismic, and remote sensing data and employs the time-series InSAR technique, slope unit segmentation, and machine learning XGBoost algorithm to identify potential landslides and to conduct LSE in the development region in Chaya County, Tibet. This region is characterized by intense neotectonic movement [60], developed faults [61], steep topography, fragile geological environment [62], and diverse human engineering activities and suffers from frequent landslide activity. Moreover, some potential landslides have not been discovered in this region because the steep relief and high elevation have brought great difficulties to field survey. Thus, the region is selected as the study area in this work. Furthermore, the cause of landslide high-susceptibility, including the function of geology, topography, hydrology, meteorology, earthquake, and human activity, are illuminated. The proposed synthetic criteria for hidden landslide identification and the LSE idea of combining potential and historical landslides can also be applied to other disaster-intensive areas.

## 2. Study Area

**Figure 1:**
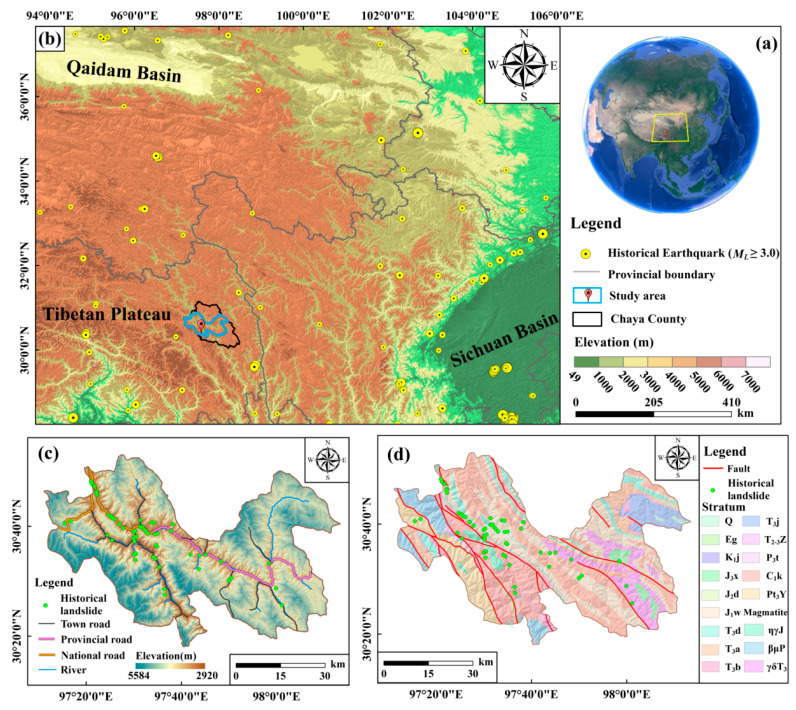
Geological and seismotectonic setting of the study area. (**a**) Location of the study area in China. (**b**) Location of the study area in the Tibetan Plateau. (**c**) Main roads, developed rivers, and historical landslides in the study area. (**d**) Strata and fault tectonics in the study area (1:200,000 geological map). The symbols include: Q = Quaternary; Eg = Gongjue Formation of Paleogene age; K_1_j = Jingxing Formation of Early Cretaceous age; J_3_x = Xiaosuoka Formation of Late Jurassic age; J_2_d = Dongdaqiao Formation of Middle Jurassic age; J_1_w = Wangbu Formation of Early Jurassic age; T_3_d = Duogaila Formation of Late Triassic age; T_3_a = Adula Formation of Late Triassic age; T_3_b = Bolila Formation of Late Triassic age; T_3_j = Jiapeila Formation of Late Triassic age; T_2–3_Z = Zhuka Formation of Middle and Late Triassic age; P_3_t = Tuobei Formation of Late Permian age; C_1_k = Kagong rock group of Early Carboniferous age; Pt_3_Y = Jiuxi Formation of Proterozoic era; ηγJ = Monzonitic granite of Jurassic age; γδT_3_ = Granodiorite of Late Triassic age; and βμP = Dolerite of Permian age. The Earth image is sourced from Google Earth.

The study area (Figure 1) consists of the development areas of Jitang Town, Yanduo Town, Xiangdui Town, Rongzhou Township, and Kagong Township in Chaya County, Tibet and covers an area of 3380.73 km^2^. The region is situated in the India-Eurasia subduction collision zone, featuring intensive crust uplift [60] and relative frequent earthquakes that were mainly reflected in the succession activities of old faults [63]. The area is characterized by complicated tectonics and developed faults and is dominated by brittle fractures with the overall tectonic trend from the northwest to southeast (1:200,000 geological map; [61]). Strata from the Proterozoic to Cenozoic are exposed, including the strata in Neoproterozoic (Pt), Carboniferous age (C), Permian age (P), Triassic age (T), Jurassic age (J), Cretaceous age (K), Paleogene age (E), and Quaternary age (Q) (1:200,000 geological map; [62]). Lithology is dominant in the rock group of weak mudstone and shale and the rock assemblage of quartz sandstone, siltstone, and volcanics [62].

A large number of mountains are distributed in the study area featuring high mountains, deep valleys, and rugged topography, i.e., a deep-cutting alpine and gorge landform. The high elevation is a remarkable feature that changes from 2920 to 5584 m, with the average of 4254 m, and the lowest position is situated in the outlet of the Lancang river. The region is typical of plateau temperate semi-arid monsoon climate [64], and rainfall concentrates from June to September. Water systems are developed, and the Lancang, Maiqu, Wangbuqu, Sequ rivers run through the area. Along both sides of the rivers, many slopes were excavated due to road construction [62]. Human engineering activity is diverse and mainly composed of slope cutting and house building, road construction, hydropower construction, mining activity, and agricultural activity [62]. The national roads G214 and G349 and the provincial highways S203 and S303 wind through the region.

Therefore, the study area is depicted by intensive crust movement [60], developed fault tectonics [61], fragile geological environment, highly weathered rocks [62], deep-cutting alpine and gorge landform, seasonal rainfall, developed drainage system, and diverse human engineering activity, which has created favorable conditions for landslide occurrence and development.

## 3. Data and Methods

### 3.1. Data

#### 3.1.1. Historical Landslide Inventory

A specific survey on geological hazards has been conducted in Chaya County at a scale of 1:50,000 by Bureau of Geology and Mineral Exploration and Development of Tibet Autonomous Region. Some landslides in Chaya County are characterized by large scales, high elevations, deeply cut surfaces, and deep sliding surfaces [65]. The historical landslide inventory is provided by Bureau of Geology and Mineral Exploration and Development of Tibet Autonomous Region. Fifty-nine historical landslides occurred in the study area, and the main disaster-controlling and disaster-triggering characteristics are shown in Supplementary Figure S1. These characteristics provide a basis for the identification criteria of potential landslides. With regard to the geological characteristics (Supplementary Figure S1a), these landslides mainly occurred in the engineering rock group of weak mudstone and shale and in the rock assemblage of relatively hard quartz sandstone, siltstone, and volcanics. Landslides also developed in the rock group of relatively hard sandstone and limestone and in the rock assemblage of weak sandstone, slate, and conglomerate. These rock groups are characterized by developed joints and severe weathering and thus contribute to landslide development [62]. In addition, 64.4% of landslides were situated within 3 km of the faults (Supplementary Figure S1b) due to the fractured rock mass and developmental fissures in the vicinity of the faults and shear zones [66,67]. Moreover, the fault strike controlled the path of rivers and the direction of valleys and influenced river erosion and terrain incision to the slopes [68,69]; thus, faults have played an important role in landslide occurrence in the study area [62]. Regarding the topographic features, landslides were concentrated in the regions with elevations of 3000–4000 m (Supplementary Figure S1c) and were mainly situated on the slopes with slope angles of 20°–30°. Moreover, all the landslides featured the slope angle larger than 10° (Supplementary Figure S1d). As for the environmental characteristics, river scouring and erosion and groundwater level fluctuation had a critical impact on landslide occurrence, and 67.8% of landslides were distributed within 500 m of the rivers (Supplementary Figure S1e). As to the disaster-triggering mechanism, landslide occurrence in the study area was closely associated with earthquake events; for example, *M*_s_ 6.1 Changdu earthquake on 12 August 2013, triggered new landslides in the study area [62]. In addition, 72.73% of landslides were induced during the rainy season from June to September, and 77.97% occurred in the region with high cumulative rainfall of 1200–1300 mm from 23 April 2018, to 26 December 2019 (Supplementary Figure S1f). Furthermore, 64.41% landslides were located within 500 m of the roads and 47.46% were within 200 m of the roads (Supplementary Figure S1g).

#### 3.1.2. Multisource Data

Eight sets of multisource data (Table 1) are adopted in this work to identify potential landslides and to conduct LSE. (1) Sentinel-1A SAR images are used to detect surface deformation based on SBAS-InSAR technique. Forty-nine ascending SAR images are employed to extract deformation displacement and velocity. (2) Setinel-2 multispectral images are adopted to extract normalized differential vegetation index (NDVI) and land use. (3) Google Earth images are employed to interpret and refine road networks and water systems. Google Earth images and Mapbox images are used to extract the micro-geomorphological and macroscopical deformation characteristics of landslides, including trailing edges, front edges, cracks, collapses, terrace scarps, gullies and so on. (4) SRTM DEM data are adopted to establish the topographic factors, including elevation, slope angle, aspect, curvature, surface roughness, surface cutting depth, relief amplitude, elevation gradient, topographic wetness index (TWI). (5) Geological maps are used to construct the geological factors consisting of strata (lithology) and distance to fault. (6) Geographic data of road networks and drainage systems, refined by Google Earth images, are adopted to extract the factors of distance to road and distance to river. (7) Seismic data with the magnitude above 3.0 and within 400 km of the study area are used to establish the seismic factors of peak ground acceleration (PGA) and kernel density of earthquake distribution that reflects the distribution concentration of earthquake events. (8) CHIRPS rainfall data are employed to build the meteorological factor of cumulative rainfall.

Therefore, according to the surface deformation, geoenvironmental, seismic, rainfall, and human engineering activity characteristics derived from the multisource data, potential landslides can be identified, and landslide susceptibility can be evaluated.

### 3.2. Methods

**Figure 2:**
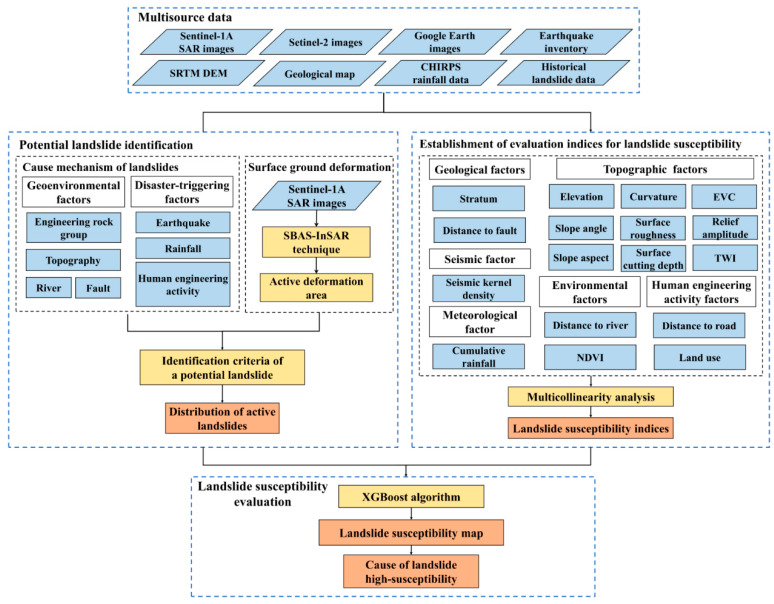
Technical route of potential landslide identification and landslide susceptibility evaluation.

The technical route is shown in Figure 2. First, the geoenvironmental factors and disaster-triggering factors are derived from the multisource data of geology, topography, geography, meteorology, earthquake, and remote sensing data. These factors consist of two classes of indices: the identification indices of potential landslides and the evaluation indices of landslide susceptibility. Second, the surface deformation is extracted from Setinel-1 SAR images. Third, potential landslides are recognized by the characteristics of surface deformation, lithology, fault tectonics, topography, micro-geomorphology, water system, earthquake, rainfall, and human engineering activity. Specifically, potential landslides are identified based on the ground deformation, disaster-controlling, and disaster-inducing indices of deformation velocity, engineering rock group, distance to fault, slope angle, landslide micro-geomorphology, distance to river, PGA, cumulative rainfall, and distance to road. Fourth, landslide susceptibility is evaluated by combining potential landslides and historical landslides based on the LSE indices via slope unit segmentation and XGBoost algorithms. The LSE map generated from the XGBoost algorithm is compared to the ones produced from the support vector machine (SVM) and convolutional neural network (CNN) algorithms. Fifth, the cause of landslide high-susceptibility, including the action of geology, topography, meteorology, and human activity, are illuminated.

#### 3.2.1. Establishment of Geoenvironmental and Disaster-Inducing Factors

According to the cause of landslide development in the study area (Section 3.1.1), two types of influencing factors related to landslide development and occurrence are established, including disaster-controlling factors and disaster-triggering factors (Table 2). All the factors, except PGA, are employed as the initial evaluation indices of landslide susceptibility. The factors of engineering rock group, distance to fault, slope angle, distance to river, PGA, cumulative rainfall, and distance to road are adopted as the identification indices of potential landslides.

The disaster-controlling factors are composed of the geological, topographic, environmental characteristics that control landslide occurrence and development. The disaster-triggering factors consist of the meteorological, seismic, and human engineering activity features that induce landslides.

#### 3.2.2. Surface Deformation Observed by SBAS-InSAR Technique

SBAS-InSAR technique [70] selects multiple main images to generate small-baseline image sets and conduct interference. The minimum norm criterion and singular value decomposition (SVD) method are employed to connect various image sets and extract surface deformation [71]. The short time and space baselines in each image set ensure the coherence of differential interference image pairs, increase the time sampling rate and spatial density of coherence, minimize the error caused by atmospheric phase delay, and guarantee the accuracy and continuity of surface deformation measurements [70].

Suppose in the study area, *N* SAR images were shot at the *N* times *t*_0_, *t*_1_, ⋯, *t_N-1_*, respectively, and *M* ($\frac{N}{2}\leq M\leq (N-1)\frac{N}{2}$) interference image pairs are generated. The differential interference phase $\delta \varphi_{k}(x,y)$ of the *k*th differential interferogram in Pixel (*x*, *y*) is generated from the SAR images shot at the times *t_a_* and *t_b_* (*t_a_* < *t_b_*) and is calculated in Equation (1) [70,72,73].

(1) $$\begin{array}{l} \delta \varphi_{k}(x,y)=\delta \varphi_{k,t_{b}}(x,y)-\delta \varphi_{k,t_{a}}(x,y)\\ =\frac{4\pi }{\lambda }[d_{k,t_{b}}(x,y)-d_{k,t_{a}}(x,y)]+\delta \varphi_{k,topo}(x,y)+\delta \varphi_{k,atom}(x,y)+\delta \varphi_{k,noi}(x,y) \end{array}$$

in which $\delta \varphi_{k,t_{a}}(x,y)$ and $\delta \varphi_{k,t_{b}}(x,y)$ are the differential interference phases at the times *t_a_* and *t_b_*, respectively; $d_{k,t_{a}}(x,y)$ and $d_{k,t_{b}}(x,y)$ are the displacements in the SAR line of sight (LOS) at the times *t_a_* and *t_b_*, respectively, which is relative to the displacement at the initial time *t*_0_; $\delta \varphi_{k,topo}(x,y)$, $\delta \varphi_{k,atom}(x,y)$, and $\delta \varphi_{k,noi}(x,y)$ are the phases of topography, atmosphere, and noise, respectively; and λ represents the sensor wavelength.

Physically, a phase is the product of velocity and time; thus, $\delta \varphi_{k}(x,y)$ can be expressed in Equation (2) [70,72].

(2) $$\delta \varphi_{k}(x,y)=\sum_{i=t_{a}+1}^{t_{b}}(t_{i}-t_{i-1})v_{i}$$

in which *v_i_* is the deformation velocity at the time *t_i_*.

All the unwrapping phases are expressed in Equation (3), in which *A* is a *M* × *N* matrix [70,72]. The SVD algorithm is used to calculate Equation (3) and to obtain the least square solution of the velocity *V* in the minimum norm sense [70,72]. Then, atmospheric and noise phases are removed by high-frequency and low-frequency filtering, and the displacement is calculated according to the integral of velocity over time [70,72]. Finally, geocoding is carried out to acquire surface deformation measurements [70,72].

(3) δφ=A⋅V

Moreover, R-index [74,75] is employed to quantitatively evaluate the visibility of deformation in LOS due to the special high and steep terrain in the study area. According to the topography, incident angle of sight, and satellite azimuth, the study area is divided into five visibility classes: good visibility, low sensitivity, foreshortening, layover, and shaded regions. The foreshortening and layover regions are characterized by geometric distortion, and the deformation in these regions cannot be effectively observed by the satellite. The deformation in the shaded areas is completely undetectable. The good visibility and low sensitivity areas are both attributed to visible areas. The surface deformation in the good visibility and low sensitivity regions can be detected by the InSAR technique without geometric distortion; thus, the two classes of regions are adopted to mask the SBAS-InSAR deformation measurements and to extract the effective deformation.

#### 3.2.3. Identification Criteria of Potential Landslides

**Figure 3:**
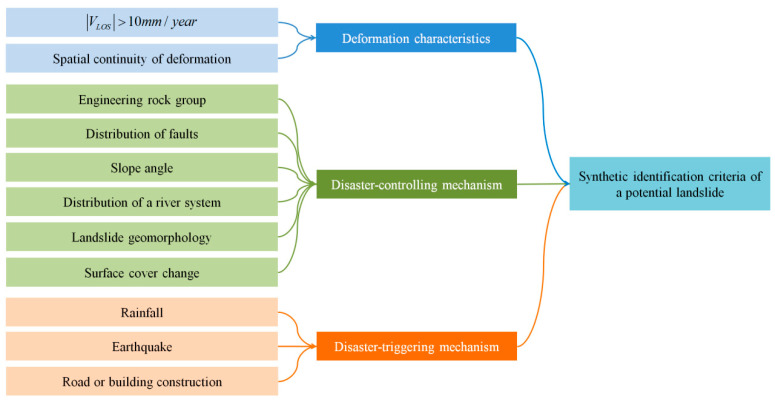
Synthetic criteria for potential landslide identification.

According to the cause of landslides in the study area (Section 3.1.1), new synthetic criteria for potential landslide identification are suggested, which consist of surface deformation, disaster-controlling, and disaster-inducing characteristics. Disaster-controlling characteristics include engineering rock groups, fault tectonics, topography, micro-geomorphology, and drainage systems. Disaster-inducing features are composed of earthquakes, rainfall, and human engineering activity. A slope is a moving potential landslide if it meets the following 8 criteria (Figure 3).

(1) The LOS deformation velocity *V*_LOS_ of the slope is larger than two times the standard deviation σ of the Setinel-1 LOS deformation velocity [18,76], and the value of σ is 5 mm/year [18]. The standard deviation indicates the uncertainty of a velocity value [76]; thus, a slope is confidently moving if $\left|V_{LOS}\right|$ >10 mm/year.
(2) The surface deformation of the slope features spatial continuity. The deformation is spatially continuous when a minimum of 2 × 2 adjacent pixels have velocities more than 2σ [77].
(3) The lithology is characterized by soft rocks or highly weathered and fractured hard rocks. In the study area, the four classes of rock groups make for landslide development: the rock group of weak mudstone and shale, the rock assemblage of relatively hard quartz sandstone, siltstone, and volcanics, the rock group of relatively hard sandstone and limestone, and the rock assemblage of weak sandstone, slate, and conglomerate. In other types of engineering rock groups, no landslide occurred in the past.
(4) The slopes within 3 km of faults feature a high occurrence rate of landslides. The rock mass appears more broken when it is closer to the fault, which avails landslide occurrence [62].
(5) The slope angle is larger than 10°.
(6) The slopes within 500 m of rivers are typical of a high frequency of landslide occurrence. The impacts of water erosion, hydrostatic pressure, and hydrodynamic pressure are remarkable when slopes are close to water systems [62,78,79].
(7) The slope possesses obvious landslide micro-geomorphologic characteristics, e.g., free surfaces, gullies, cracks, slide terraces, and collapses. Moreover, the slope features surface cover changes, e.g., vegetation destruction or bare land expansion.
(8) The surface deformation of the slope is triggered by definite factors. There is a significant correlation between the deformation displacement or velocity and the variation of the inducing factors. e.g., PGA, cumulative rainfall, or distance to road or building. The correlation is quantitatively measured by the Pearson correlation coefficient passing a significance test under the significance level of 0.05 [77].

#### 3.2.4. XGBoost Algorithm for Landslide Susceptibility Evaluation

XGBoost, under the full name of Extreme Gradient Boosting [80], is a refined and distributed open-source library of Gradient Boosting techniques and is regarded as one of machine learning algorithms featuring high precisions and high computational efficiencies. XGBoost generates a strong landslide susceptibility prediction model from an ensemble of weak prediction models (decision trees) using a gradient descent algorithm; thus, it can effectively overcome overfitting [80]. It employs a stage-wise fashion and in each iteration, produces a weak prediction model by decreasing the residual and optimizing the loss function value [80]. The objective function is composed of a loss function of gradient boosting and a regularization term (Equation 4) [80]. The loss function is expanded by a second-order Taylor series and is solved by the Newton’s method [80].

(4) $$L^{\left(t\right)}=\sum_{i=1}^{n}l(y_{i},y_{i}^{\prime }{}^{\left(t-1\right)}+f_{t}(x_{i}))+\sum_{k=1}^{t}\Omega (f_{k})$$

in which *t* is the number of decision trees, *n* is sample number, *y_i_* and $y_{i}^{\prime }$ are the real and predicted values, respectively, *f_t_*(*x_i_*) is the prediction value of the *t*th decision tree, and the function *l*(·) is the loss of single sample. The regularization term is defined in Equation (5) using L2 norm and reflects the complexity of a tree [81]. The smaller is the value of Ω(f), the lower is the complexity of the tree, and then the stronger is the generalization ability of the tree.

(5) $$\Omega (f)=\gamma T+\frac{1}{2}\lambda {\left\|\omega \right\|}^{2}$$

in which *T* and *ω* are the number and weights of tree nodes, respectively, *γ* is the regularization coefficient of the number of tree nodes, and λ is the regularization coefficient of the weights of tree nodes.

In this work, a surface curvature watershed algorithm [81] is employed to segment the study area into 170,188 slope units according to the DEM data. A total of 1136 slope units, with the same number of landslide and non-landslide samples, are randomly split into 70% of training samples and 30% of testing samples to train the XGBoost model and to evaluate the model precision, respectively. Moreover, the LSE indices are inspected in multicollinearity by the two parameters of Tolerance (TOL) and Variance Inflation Faction (VIF) [82]. The indices with strong correlations are eliminated to improve the precision of LSE.

## 4. Results

### 4.1. Identification of Potential Landslides

**Figure 4:**
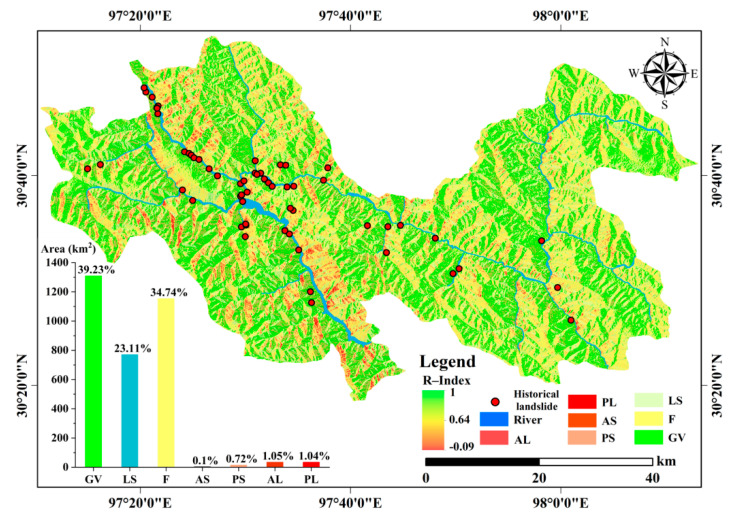
Visibility analysis by R-index. The abbreviations include: AL = active layover; PL = passive layover; AS = active shade; PS = passive shade; F = foreshortening; LS = low sensitivity; and GV = good visibility.

**Figure 5:**
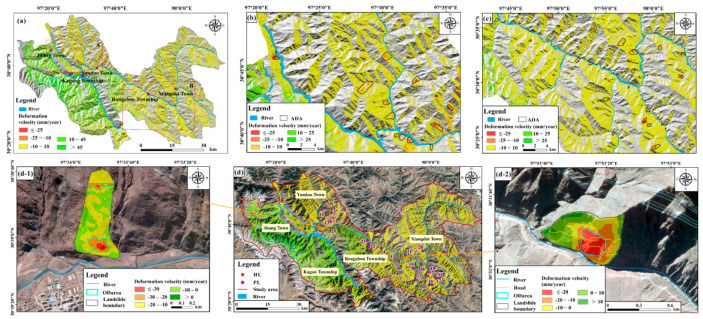
Recognition of active landslides. (**a**) Deformation velocity in the good visibility and low sensitivity regions. (**b**) Active deformation areas in Sub-region A. (**c**) Active deformation areas in Sub-region B. (**d**) 25 identified active landslides including active historical landslides and active potential landslides. (**d-1**) Deformation characteristics of the active landslide AL-25. (**d-2**) Deformation features of the newly discovered landslide AL-21. The base images are Mapbox images. The abbreviations include: ADA = Active deformation area; HL = Historical landslide; PL = Potential landslide; and ODarea = Obvious deformation area.

**Figure 6:**
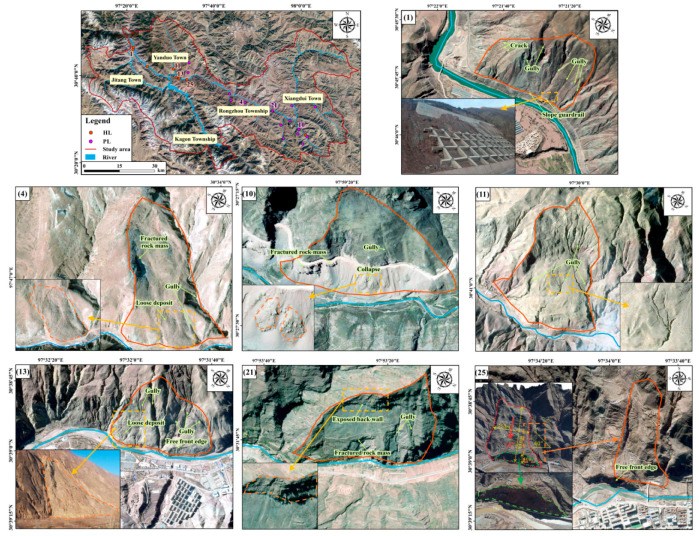
Validation of identified active landslides via field survey, UAV images, and 3D mapbox images. The field survey photos and UAV images are provided by Bureau of Geology and Mineral Exploration and Development of Tibet Autonomous Region and from the reference [62].

The visibility analysis based on R-index is shown in Figure 4. The good visibility and low sensitivity regions (visible regions), with an area of 2071.16 km^2^, occupy 62.34% of the whole study area, and 86.44% of historical landslides occurred in the two types of regions. The study area features high elevations and a deep-cutting alpine and canyon landform. The special topography and landform may cause geometric distortion in the SAR interference result. The effective deformation can be extracted only in the good visibility and low sensitivity areas without geometric distortion. Thus, the displacement measurement extracted by SBAS-InSAR technique is masked by the R-Index visibility value, and the displacement and velocity in the good visibility and low sensitivity regions are retained (Figure 5). According to the proposed synthetic criteria, 25 active landslides are identified, in which 16 ones are newly discovered as potential landslides (Figure 5). The identified active landslides are validated by field survey, 3D UAV images, and 3D mapbox images and all exhibit obvious deformation signs including cracks, collapses, gullies, fragmented rock mass and so on. Seven validation examples are shown in Figure 6, and the concrete deformation signs and features are shown in Supplementary Figures S2–S4. Moreover, the validation of 25 identified active landslides is shown in Supplementary Figure S5.

Furthermore, the superiority of the proposed criteria is illuminated in Supplementary Figures S6 and S7. There are a large number of false alarm regions generated by SBAS-InSAR technique, and some representative examples are indicated in Supplementary Figure S6. Sixty-eight false alarm areas are not identified as active landslides according to the suggested criteria, and these areas can be divided into three classes. (1) 11.76% are ground subsidence caused by human engineering activity, e.g., road construction, building construction, and mine exploitation, rather than mass movement down a slope. (2) 47.06% are actually noises due to the inherent shortcoming of InSAR technique. These fake deformations have not been related to any landslide-inducing factor and cannot reflect surface deformation. (3) 41.18% do not exhibit landslide micro-geomorphological features, without free surfaces, and some fake areas are characterized by hard intrusive rocks, e.g., monzonitic granite; thus, the topographic and lithology preconditions of landslide development are absent. Therefore, the proposed criteria can effectively reduce the false alarm.

### 4.2. Landslide Susceptibility Evaluation

**Figure 7:**
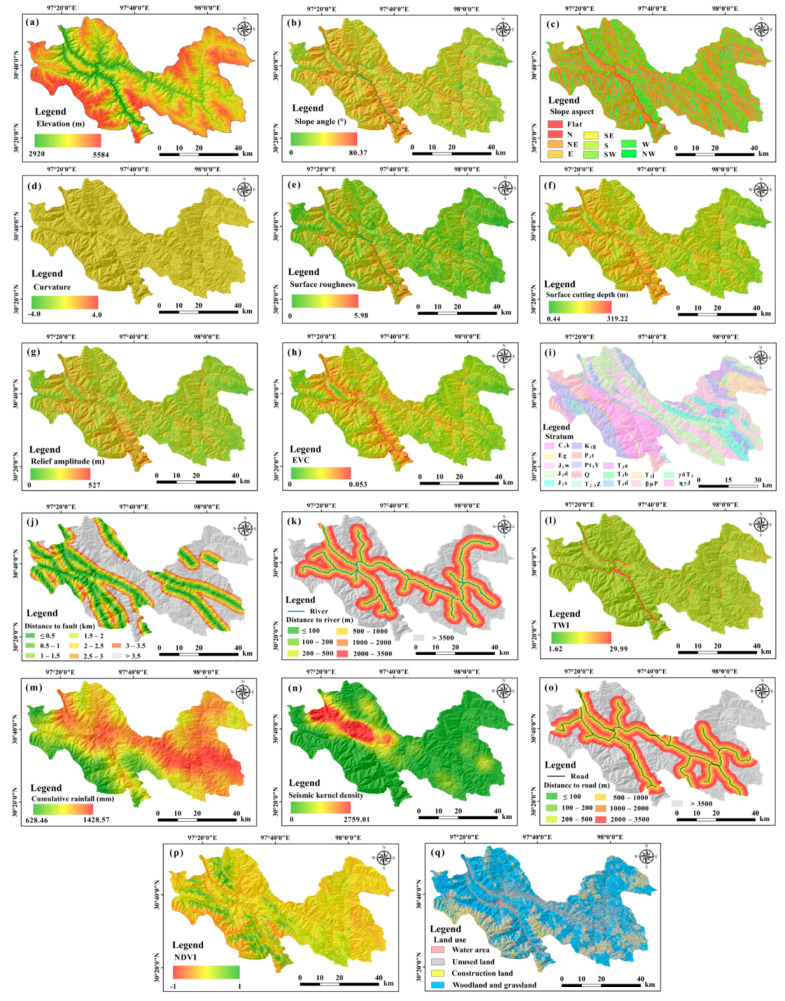
Initial indices of landslide susceptibility assessment constructed from multisource data. (**a**) Elevation. (**b**) Slope angle. (**c**) Slope aspect. (**d**) Curvature. (**e**) Surface roughness. (**f**) Surface cutting depth. (**g**) Relief amplitude. (**h**) Elevation variation coefficient (EVC). (**i**) Stratum. (**j**) Distance to fault. (**k**) Distance to river. (**l**) Topographic wetness index. (**m**) Cumulative rainfall. (**n**) Seismic kernel density. (**o**) Distance to road. (**p**) Normalized difference vegetation index. (**q**) Land use.

The initially established evaluation indices are shown in Figure 7. The multicollinearity analysis result is shown in Supplementary Table S3. There is collinearity among the factors of slope angle, surface roughness, surface cutting depth, elevation variation coefficient, and relief amplitude. As mentioned above, slope angle is very important for landslide development; thus, the other four factors of surface roughness, surface cutting depth, elevation variation coefficient, and relief amplitude are eliminated. There is no collinearity among the remained 13 factors, so they are employed as the assessment indices of landslide susceptibility.

**Figure 8:**
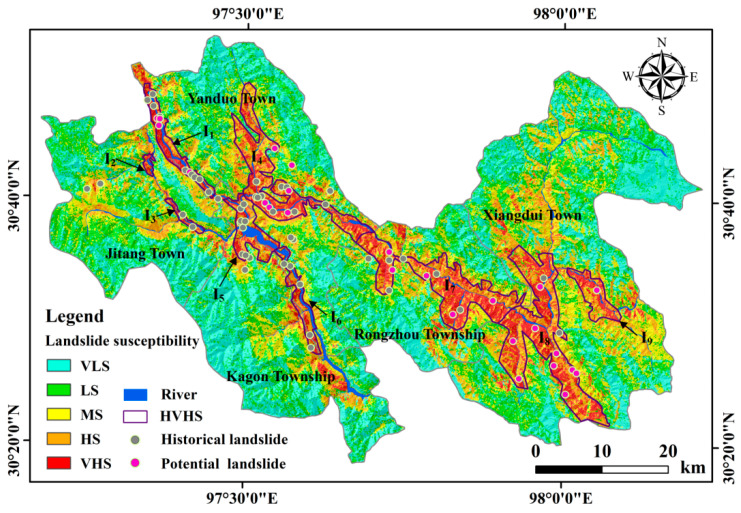
Landslide susceptibility evaluation map. The abbreviations include: VLS = Very low susceptibility; LS = Low susceptibility; MS = Medium susceptibility; HS = High susceptibility; VHS = Very high susceptibility; and HVHS = High and very high susceptibility.

The LSE map is shown in Figure 8, the susceptibility statistics is shown in Table 3, and the accuracy assessment is shown in Table 4. 92% of potential and known landslides are situated in the high and very high susceptibility regions that occupy 21.85% of the whole study area. The values of the precision indices of AUC, Accuracy, TPR, F1-score, and Kappa coefficient reach 0.996, 97.98%, 98.77%, 0.98, and 0.96, respectively.

The distribution characteristics of various susceptibility levels are illuminated as follows. The very low and low susceptibility regions are primarily located on the high or very high mountains with elevations above 4000 m, and thus human engineering activity is few in these regions. In addition, these regions mainly feature relatively flat topography with slope degrees lower than 20° and are dominated by quartzy sandstones, silty claystones, and limestones. Moreover, these areas suffer from relatively little influence from river erosion, precipitation, and earthquakes because of relatively far distances from rivers, low cumulative rainfall, and small earthquake occurrence densities. Therefore, the slopes in these regions are relatively stable.

The medium susceptibility areas are mainly situated on the high or medium-high mountains with elevations between 3500 m and 4500 m and are characterized by relatively steep relief with main slope degrees between 20° and 40°. The lithology is dominant in sandstones, claystones, slates, and siltstones with shales. Moreover, these regions are to some degree influenced by river erosion and human engineering activity, and some slopes gradually lost stability.

The high and very high susceptibility regions are primarily situated on high mountains or alpine and gorge regions with elevations lower than 4000 m. The topography is steep with slope degrees from 20° to 60°. The lithology is dominated by slates, metasandstones, phyllites, and flysch and features schistosity and mylonitization. In addition, these regions are cut by multiple faults including the Lancangjiang fault zone, Zuotongcun fault, and Chuanqiucuo fault. Moreover, these areas are seriously influenced by abundant precipitation (with cumulative rainfall larger than 1140 mm) and are relatively close to roads and rivers. Therefore, the slopes became instable and moving under the combined action of steep relief, fragmented rock mass, developed faults, active tectonic movement, abundant rainfall, intense river undercutting, and intensive human engineering activity.

### 4.3. Comparison with the SVM and CNN Algorithms

**Figure 9:**
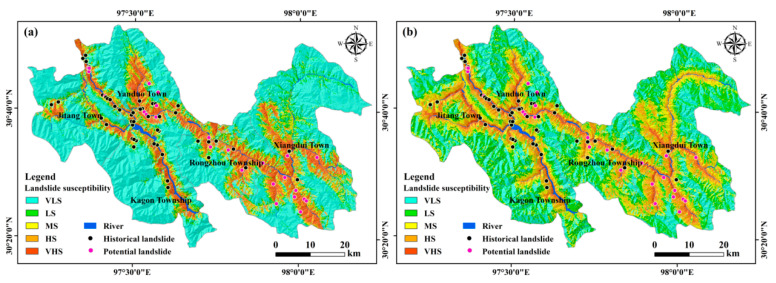
Landslide susceptibility evaluation maps generated by the (**a**) SVM and (**b**) CNN algorithms. See Figure 8 for the meaning of the legend.

**Figure 10:**
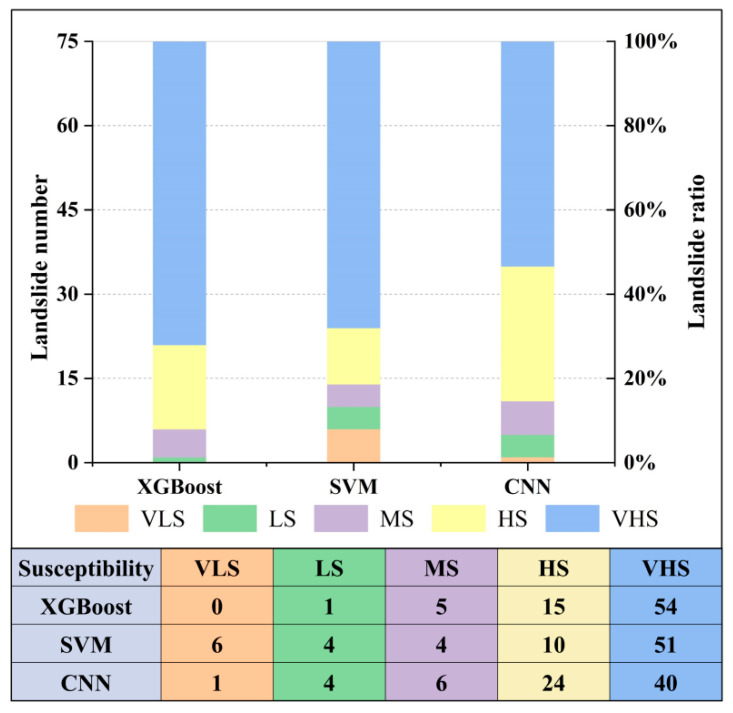
Comparison of landslide susceptibility statistics of the three algorithms. See Figure 8 for the meaning of the abbreviations.

**Figure 11:**
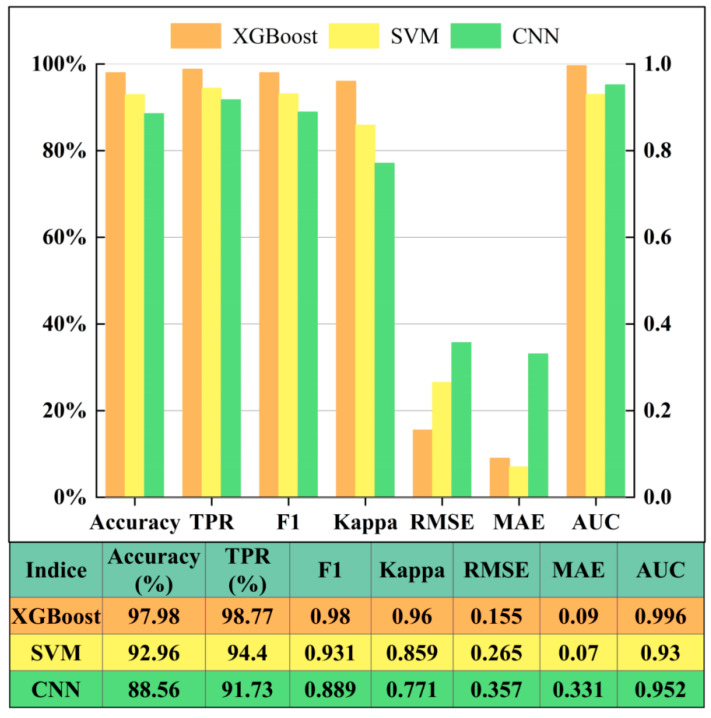
Precision comparison of landslide susceptibility assessment by the three algorithms.

SVM [83] and CNN [84] are classical machine learning and deep learning algorithms, respectively; thus, in this work, LSE results produced by the XGBoost, SVM (Figure 9a), and CNN (Figure 9b) algorithms are compared. The principle of SVM is to calculate a hyperplane and to transform linear inseparability in a low-dimensional space to linear separability in a high-dimensional space by the hyperplane [83]. In this work, radial basis functions are adopted as the kernel function, and the values of the parameters *C* and γ are set as 10 and 0.1, respectively. CNN employs convolutional operations to replace the traditional matrix multiplication operation and generally consists of an input layer, convolutional layers, active layers, pooling layers, and full connected layers [84]. In this work, two convolutional layers and two pooling layers are used to extract sematic features. The function of cross entropy [85] is adopted as the loss function, and Adam optimizer [86] is utilized to update network weight values and to facilitate convergence speed by momentum and a self-adaptative learning rate. As shown in Figure 10, the number proportions of landslides falling in the very high susceptibility regions generated by the XGBoost, SVM, and CNN algorithms are 72%, 68%, and 53%, respectively. The landslide number proportions in the high and very high susceptibility (HVHS) areas produced by the XGBoost, SVM, and CNN algorithms are 92%, 81%, and 85%, respectively. In general, XGBoost has the highest precision in various accuracy indices (Figure 11). For example, the AUC values of the LSEs by the XGBoost, SVM, and CNN algorithms are 0.996, 0.93, and 0.952, respectively, and the Accuracy values are 97.98%, 92.96%, and 88.56%, respectively. Therefore, XGBoost outperforms the other two algorithms in LSE in the study area.

### 4.4. Comparison with the LSE Map Generated from Known Landslides

**Figure 12:**
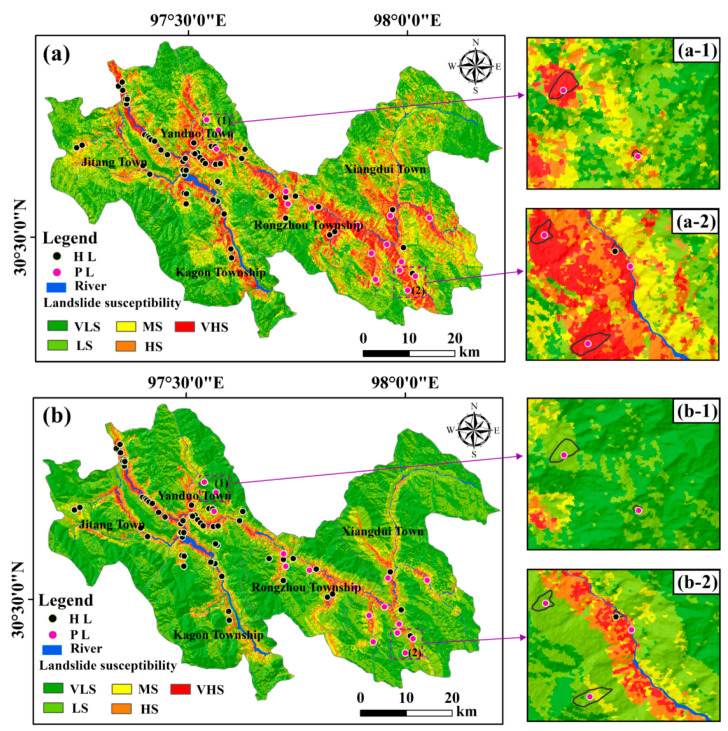
Comparison with the landslide susceptibility evaluation (LSE) only according to known landslides. (**a**) LSE map generated from known and potential landslides. (**b**) LSE map produced from known landslides. See Figure 5 and Figure 8 for the meaning of the legend.

The LSE map generated from both potential and known landslides is compared with the one produced only from known landslides (Figure 12). In the later map, 8.62% and 10.34% of historical landslides fall in the low susceptibility and medium susceptibility regions, respectively. Furthermore, in the later map, 43.75% and 6.25% of potential landslides are situated in the low susceptibility and medium susceptibility regions, respectively. Therefore, the LSE map based on known landslides cannot involve the threat from potential landslides and thus, restrict the precision and rationality of a LSE map.

## 5. Discussions

### 5.1. Cause Characteristics of Active Landslides

**Figure 13:**
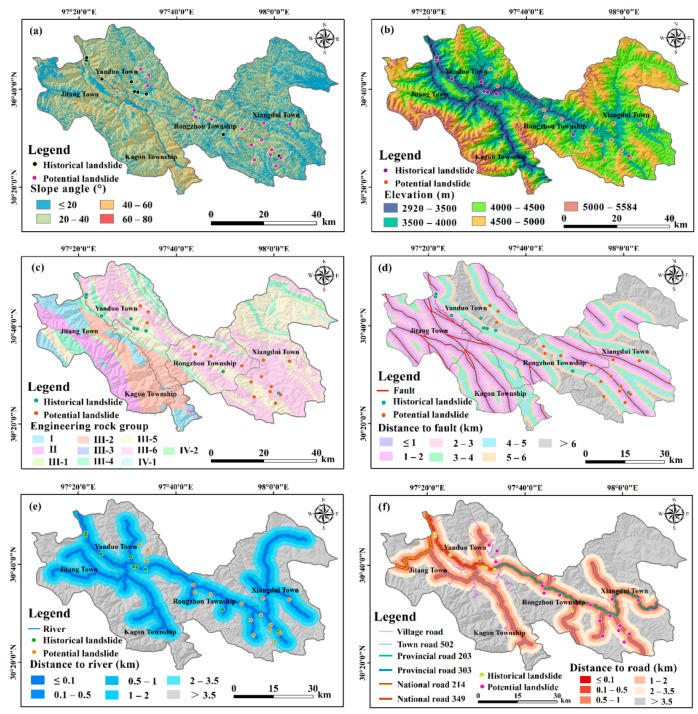
Disaster-controlling and disaster-triggering characteristics of active landslides. (**a**) Relation between active landslide distribution and slope angles. (**b**) Link between active landslide distribution and elevations. (**c**) Distribution of active landslides on engineering rock groups. (**d**) Distance from active landslides to faults. (**e**) Distance from active landslides to rivers. (**f**) Distance from active landslides to roads. See Table 2 for the meaning of engineering rock groups.

The cause characteristics of active landslides include disaster-controlling features and disaster-inducing features. The disaster-controlling characteristics of active landslides mainly include 4 aspects (Figure 13 and Table S1). (1) All the active landslides are located on the steep mountains with high elevations. The overwhelming majority occurred on the high and precipitous slopes with slope angles larger than 20° and elevations higher than 3000 m. Five landslides are situated on the slopes with slope angles larger than 30°. (2) 96% are distributed in the rock group of weak mudstone and shale or in the rock assemblage of relatively hard quartz sandstone, siltstone, and volcanics. One landslide occurred in the rock group of relatively hard sandstone and limestone. Thus, soft rocks and fractured and easily weathered hard rocks are important disaster-causing conditions in the study area. (3) 44% are situated within 2 km of the faults, 24% are within 1 km of the faults, and one landslide is directly cut by the Zuotongcun fault. Joints, fissures, and cracks are developed, rock mass is broken, and loose solid materials are deposited in the vicinity of the faults [62]. These provide abundant material sources, create a favorable condition for rainwater infiltration and physical weathering and have a remarkable influence on slope stability in the study area [62,87,88]. (4) 68% are distributed within 500 m of the rivers, 52% are within 100 m of the rivers, and 8 landslides are eroded by the rivers at the front edges. In the study area, fluvial erosion is strong, and, for example, the specific erosion values along the Lancang trunk river attain 100–500 t/km².a [62]. A number of high and steep slopes formed under strong river down-cutting and erosion, the front edges started moving, and thus pull-type landslides developed [62]. Moreover, the hydrodynamic pressure caused by groundwater seepage decreased the effective stress and shear strength on the potential sliding surface and further destroyed slope stability [62].

The disaster-inducing characteristics of active landslides are primarily embodied as the functions of earthquakes, precipitation, and human engineering activity (Table S2). In the study area, landslides triggered by human activity are closely related to road construction, e.g., the construction of National Highways G214 and G349 and Provincial Highway S203 (Figure 13) [62]. These constructions, accompanied by explosion, caused slope excavation and destroyed slope balance [62]. There are mainly three disaster-triggering features. (1) Precipitation is the foremost factor inducing active landslides in the study area. Twenty-three landslides were moving under the action of rainfall, in which 12 ones were induced mainly by rainfall, 5 by the coupled action of rainfall and road construction, 5 by the combined action of earthquakes and precipitation, and 1 by the common function of earthquakes, precipitation and human engineering activity. (2) Human engineering activity, especially road construction, plays a critical role in the development of active landslides. The deformation of 7 landslides was triggered by road construction. In addition to the 6 ones relevant to road construction mentioned in Point (1), one landslide was creeping under the coupled action of earthquakes and road construction. (3) Neotectonic movement and intensive geologic agent have a significant influence on the movement of active landslides. The deformation of 8 landslides was induced by earthquakes. Except the 7 ones induced by the combined action, one landslide was triggered primarily by earthquakes.

Therefore, in the study area, tectonic movement, developed faults, weak and easily sliding strata, highly weathered and fractured rock mass generated a mass of loose solid materials and created a large number of fissures and cracks. Then rainwater infiltrated into the cracks [89,90], rivers eroded the front edge, groundwater fluctuated to generate hydrodynamic pressure, and slope feet were excavated and exploded under road construction. Thus, the shear strength decreased, the weak sliding surface formed, the slope balance was destroyed, and the slopes became to move and creep down the high and steep topography and developed into an active landslide.

### 5.2. Cause of Landslide High-Susceptibility

**Figure 14:**
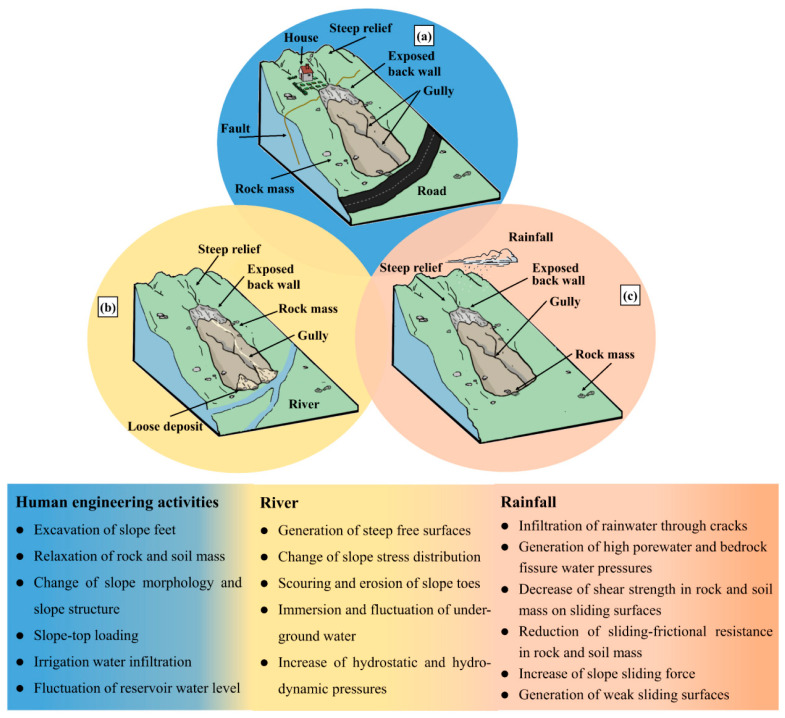
Cause of landslide high-susceptibility in the study area.

Geographically, the HVHS regions can be divided into three sub-regions (Figure 8): the area in Jitang and Yanduo Towns (I1, I2, I3, and I4 regions), the common boundary area of Kagong Township and Yanduo Town (I5 and I6 regions), and the region in Rongzhou Township and Xiangdui Town (I7, I8, and I9 regions). The cause of landslide high-susceptibility in the study area is shown in Figure 14.

According to field investigation, the HVHS area in Jitang and Yanduo Towns is situated in the river valley deeply cut by the Lancang river or in the downstream of the Maiqu river. Thus, a large amount of high and steep slopes were generated under the strong river erosion. Sandstone, siltstone, mudstone, and limestone were exposed in the region [62]. The lithology features poor mechanical property, weak structural surfaces, and secondary fissures [62]. The Lancangjiang fault zone traversed the region, featuring the fault fracture zone in a width of 150–200 m and abundant cataclasites [62]. Loose colluvial and proluvial deposits were distributed along two sides of the river valleys [62]. Moreover, the HVHS area is located along the Chaya sections of National Highway G214 and Provincial Highway S203, and this area is characterized by the most frequent and concentrated human activities in Chaya County. The construction of the highway S203 cut and exploded the slopes and destroyed slope stability. Therefore, the HVHS region was generated by the combined action of a developed fault zone, fractured rock mass, high and steep topography, strong river erosion, and frequent human engineering activity.

The HVHS area in the common boundary region of Kagong Township and Yanduo Town is distributed in the river valley deeply cut by the Lancang river, and the slope feet were scoured by the Lancang and Sequ rivers to loose stability. The area features developed faults, e.g., the Lancangjiang fault zone and Shela fault, and abundant loose residual sediments were generated due to seriously ruptured rock mass, developed cracks and cleavages, and intensive weathering [62]. Furthermore, the human engineering activity in the region included road construction, house building, hydropower construction, and farmland cultivation and irrigation. Thus, the HVHS area was caused by the common function of developed faults, broken rock mass, loose deposits, fluvial abrasion, and human engineering activity.

The HVHS region in Rongzhou Township and Xiangdui Town is located along the Maiqu river and its tributaries. The lithology was mainly composed of sandstone, siltstone, and mudstone, and the Chuanqiucuo and Zuotongcun faults spread over the area [62]. A mass of loose deposits were developed due to fractured rock mass and strong weathering [62], and high and steep slopes prevailed in the region. Surface runoff generated by rainfall converged into channels and led to intensive washing on the slope feet. The human engineering activity primarily consisted of road construction, hydropower development, agricultural activity, and house building. Therefore, the HVHS region was generated under the coupling function of soft rocks, fault tectonics, broken rock mass, loose solid deposits, alpine canyon landform, river deep-cutting, precipitation, and human activity.

## 6. Conclusions

LSE is a vital and effective technique for the prediction, prevention, and control of catastrophic landslides and becomes an essential support to the sustainable development of nations and society. Moreover, about 80% of disastrous landslides have not been discovered in advance [8], and the discovery of these hidden landslides has become a worldwide challenge due to good concealment and high and steep relief. This work proposes new synthetic criteria to improve the identification accuracy of potential landslides, and the criteria include surface deformation, disaster-controlling features, and disaster-inducing characteristics. Furthermore, this work integrates historical landslides and potential landslides to improve the precision and rationality of LSE. The synthetic criteria of potential landslide identification and the idea of combining known landslides and potential landslides can be utilized to other disaster-serious regions.

This work selects Chaya County, a representative region significantly threatened by landslides, as the study area and employs multisource data (geological, topographical, geographical, hydrological, meteorological, seismic, and remote sensing data) to identify potential landslides and realize LSE by the SBAS-InSAR technique and XGBoost algorithm. Three main conclusions are drawn as follows.

(1) The proposed synthetic criteria integrate the characteristics of deformation, geology, topography, geomorphology, environment, earthquake, rainfall, and human engineering activity. According to the criteria, 25 active landslides are identified, among which 16 ones are newly discovered as potential landslides. In the study area, tectonic movement, weak strata, and fractured rock mass generated abundant cleavages and cracks and created numerous loose deposits that tended to move down the steep slopes under the action of external forces. Under the coupled function of strong river erosion, earthquake ground motion, rainwater infiltration, hydrodynamic pressure, and road and building construction, the shear strength decreased, the slope became moving, and an active landslide occurred.
(2) A LSE map is generated by slope unit segmentation and the XGBoost algorithm. 92% of the potential and known landslides are situated in the HVHS regions that occupy 21.85% of the whole study area. The values of the precision indices of AUC, Accuracy, TPR, F1-score, and Kappa coefficient reach 0.996, 97.98%, 98.77%, 0.98, and 0.96, respectively. Moreover, XGBoost outperforms the representative machine learning algorithm of SVM and the deep learning algorithm of CNN in the study area.
(3) The HVHS region is situated in the river valley, suffering from strong river erosion, and features high and steep topography. The region was cut by various faults, and a large amount of cataclasites and loose deposits were generated and are distributed along two sides of the river valleys. Rainwater washed the slope feet and penetrated through the loose soils and broken rocks into the slope bodies. Moreover, the slope feet in the HVHS region were relaxed and excavated by the construction of the national or provincial highways. Therefore, the HVHS was caused by the coupled action of a developed fault zone, ruptured rock mass, high and steep relief, intensive river erosion, concentrated rainfall, and frequent human engineering activity.

The future research can be conducted on 2 aspects: (1) automatic identification of active landslides over an extensive region, and (2) an implication of landslide susceptibility on seismic hazard assessment. First, in this work, active landslides are recognized in an interactive computer-aided mode. There may be numerous active landslides occurring over a wide region; thus, automatic recognition of active landslides can to a great degree improve the identification efficiency and provide an effective support for landslide prevention and control. Second, ground motion prediction has been a significant technique for seismic hazard and risk assessment and provides a crucial clue on earthquake risk mitigation [91]. In a region with intensive crustal movement, a landslide susceptibility map can be employed to predict the permanent ground displacement (PGD) triggered by an earthquake [2]. The PGD, combined with the transient ground displacement, is a vital basis for earthquake risk evaluation and control [2,91].

## Figures and Tables

**Table 1:** Multisource data adopted in this work. The abbreviations include: USGS = United States Geological Survey; CHIRPS = Climate Hazards Group infraRed Precipitation with Station data; SRTM = Shuttle Radar Topography Mission; and UCSB = University of California Santa Barbara.

Data Type	Data	Date	Resolution	Data Source
Image	Sentinel-1A SAR image	23 April 2018–26 December 2019	5 m × 20 m	European Space Agency
	Google Earth image	7 February 2015, 16 March 2015, 15 June 2021	0.38 m, 0.31 m	Google Earth
	Setinel-2 image	18 October 2019	10 m	European Space Agency
	Mapbox image	28 December 2019	0.51 m	SAS.Planet
Topography	SRTM DEM	2000	30 m	USGS
Geology	Geological map	—	1:200,000	National Geological Archives
Geography	Road network	2017	—	National Catalogue Service for Geographic Information, Google Earth images
	Water system	2017	—	
Seismology	Earthquake inventory	April 2018–December 2019	—	China Earthquake Administration China Earthquake Networks Center
Meteorology	CHIRPS Satellite	23 April 2018–26 December 2019	5 km	UCSB

**Table 2:** Geoenvironmental and disaster-inducing factors of landslides in the study area.

Factor Type		No.	Factor	Grade
Geoenvironmental factor	Topographic	1	Slope aspect *	(1) Flat; (2) N; (3) NE; (4) E; (5) SE; (6) S; (7) SW; (8) W; (9) NW
		2	Slope angle (°) *	Continuous
		3	Curvature *	Continuous
		4	Elevation (m) *	Continuous
		5	Surface roughness	Continuous
		6	Surface cutting depth (m)	Continuous
		7	Relief amplitude (m)	Continuous
		8	EVC	Continuous
		9	TWI *	Continuous
	Geological	10	Stratum *	(1) Q; (2) Eg; (3) K_1_j; (4) J_3_x; (5) J_2_d; (6) J_1_w; (7) T_3_d; (8) T_3_a; (9) T_3_b; (10) T_3_j; (11) T_2–3_Z; (12) P_3_t; (13) C_1_k; (14) Pt_3_Y; (15) ηγJ; (16) βμP; (17) γδT_3_
		11	Engineering rock group	(1) I; (2) II; (3) III-1; (4) III-2; (5) III-3; (6) III-4; (7) III-5; (8) III-6; (9) IV-1; (10) IV-2
	Tectonic	12	Distance to fault (km) *	(1) ≤0.5; (2) 0.5–1; (3) 1–1.5; (4) 1.5–2; (5) 2–2.5; (6) 2.5–3; (7) 3–3.5; (8) >3.5
	Environmental	13	Distance to river (m) *	(1) ≤100; (2) 100–200; (3) 200–500; (4) 500–1000; (5) 1000–2000; (6) 2000–3500; (7) >3500
		14	NDVI *	Continuous
Triggering factor	Meteorological	15	Cumulative rainfall (mm) *	Continuous
	Seismic	16	PGA (g)	Continuous
		17	Kernel density *	Continuous
	Human engineering activity	18	Distance to road (m) *	(1) ≤100; (2) 100–200; (3) 200–500; (4) 500–1000; (5) 1000–2000; (6) 2000–3500; (7) >3500
		19	Land use *	(1) Construction land; (2) Woodland and grassland; (3) Water area; (4) Unused land

The factors marked by “*” indicate the ones that pass the inspection of multicollinearity and are utilized in landslide susceptibility evaluation. The abbreviations include: EVC = Elevation variation coefficient; TWI = Topographic wetness index; PGA = peak ground acceleration; NDVI = Normalized difference vegetation index; I = Hard intrusive rock series; II = Rock group of relatively weak schist and gneiss; III-1 = Rock assemblage of relatively hard limestone, quartz sandstone, and volcanics; III-2 = Rock assemblage of weak sandstone, slate, and conglomerate; III-3 = Rock group of relatively weak volcanics and clasolite; III-4 = Rock series of relatively hard sandstone and limestone; III-5 = Rock group of weak mudstone and shale; III-6 = Rock assemblage of relatively hard quartz sandstone, siltstone, and volcanics; IV-1 = Rock group of loose ice water deposit boulder, gravel, and clay; IV-2 = Rock assemblage of loose alluvial-diluvial sand, gravel, and clay. See Figure 1 for the meaning of the stratum symbols.

**Table 3:** Landslide susceptibility statistics.

Indice	Very Low Susceptibility	Low Susceptibility	Medium Susceptibility	High Susceptibility	Very High Susceptibility
Area (km^2^)	987.63	1003.59	595.07	442.49	280.73
Area proportion (%)	29.84	30.33	17.98	13.37	8.48
Landslide number	0	1	5	15	54
Landslide number proportion (%)	0	1.33	6.67	20	72

**Table 4:** Precision evaluation of landslide susceptibility assessment. TPR is the true positive rate that is equal to the recall rate, MAE indicates the mean absolute error, RMSE is the root mean squared error, and AUC is the area under the ROC (receiver operating characteristic curve).

Accuracy (%)	TPR (%)	F1-Score	Kappa Coefficient	RMSE	MAE	AUC
97.98	98.77	0.98	0.96	0.155	0.09	0.996

## Data Availability

All data are available within this article and its Supplementary Material.

## Supplementary Materials

The following supporting information can be downloaded at: https://www.mdpi.com/article/10.3390/ijerph192114241/s1, Figure S1: Distribution characteristics of the historical landslides in the study area; Figure S2: Deformation characteristics and triggering factors of AL-1 Landslide; Figure S3: Deformation monitoring and inducing factors of AL-21 landslide; Figure S4: Macroscopical deformation signs of two potential landslide examples (AL-10 and AL-20); Figure S5: Validation of 25 identified active landslides via field survey [62], UAV images [62], and 3D Mapbox images; Figure S6: False alarm regions generated by SBAS-InSAR technique superimposed on (a) Deformation velocity map and (b) Google Earth images shot on 2 February 2015; 16 March 2015; 7 November 2020; and 11 November 2020, respectively; Figure S7: Three examples of false alarms; Table S1: Geoenvironmental features of 25 active landslides; Table S2: Correlation of active landslide deformation with various triggering factors; Table S3 Collinearity relation among various disaster-controlling and disaster-triggering factors.
