# Supplementary material for: Landslide Susceptibility Evaluation Based on Potential Disaster Identification and Ensemble Learning
Source: Int J Environ Res Public Health. 2022 Oct 31;19(21):14241. doi: 10.3390/ijerph192114241 (PMC9656294; doi:10.3390/ijerph192114241)
Supplement: Supplementary file 1 [file ijerph-19-14241-s001.zip › ijerph-1959211- Supplementary Material.pdf]

Landslide susceptibility evaluation based on potential disaster identification and ensemble learning

Xianmin Wang[1], Xinlong Zhang[1], Jia Bi[1], Xudong Zhang[2, 3], Shiqiang Deng[3], Zhiwei Liu[1], Lizhe Wang[1], Haixiang Guo[1]*

[1]Hubei Subsurface Multi-scale Imaging Key Laboratory, School of Geophysics and Geomatics, Key Laboratory of Geological and Evaluation of Ministry of Education, School of Economics and Management, China University of Geosciences, Wuhan, 430074, China

[2]Institute of Geological Survey of Tibet Autonomous Region, Lhasa, 850000, China

[3]The Fifth Geological Brigade, Bureau of Geology and Mineral Exploration and Development of Tibet Autonomous Region, Glomud, 816000, China

xianminwang@163.com

Online Resource 1: Cause characteristics of historical landslides

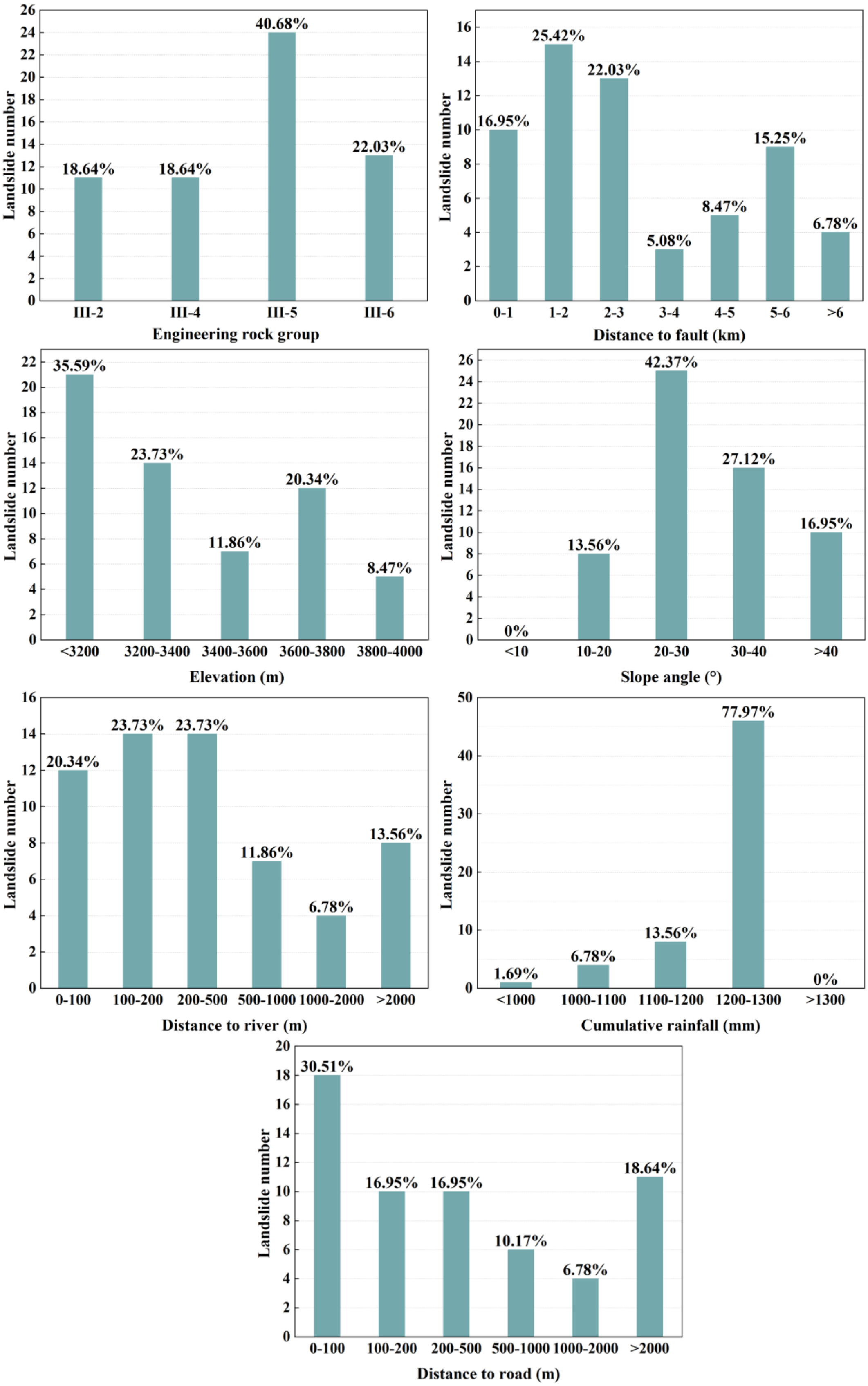

Supplementary Figure S1 Distribution characteristics of the historical landslides in the study area. (a) Relation between landslides and engineering rock groups. (b) Distance from landslides to faults. (c) Link between landslides and elevation. (d) Relationship between landslides and slope angles. (e) Distance from landslides to drainage systems. (f) Relation between landslides and cumulative rainfall from April 23, 2018 to December 26, 2019. (g) Distance from landslides to main roads. The symbols of engineering rock groups include: III-2=Rock assemblage of weak sandstone, slate, and conglomerate; III-4=Rock series of relatively hard sandstone and limestone; III-5=Rock group of weak mudstone and shale; and III-6=Rock assemblage of relatively hard quartz sandstone, siltstone, and volcanics.

Online Resource 2: Geoenvironmental and triggering features of active landslides

Supplementary Table S1 Geoenvironmental features of 25 active landslides. Slope angle and elevation mean the average slope angle and average elevation of a landslide. If roads are constructed on a landslide body, the distance of the landslide to roads is set to 0. See Table 2 for the meaning of engineering rock groups.

No.	Type	Area (km^2)	Slope angle (°)	Elevation (m)	Engineering rock group	Distance to fault (km)	Distance to river (km)	Distance to road (km)
AL-1	Known	0.29	40.57	3324.61	III-5	1.42	0.24	0.00
AL-2	Known	0.01	26.59	3032.82	III-6	1.42	0.07	0.00
AL-3	Known	0.02	21.38	3101.44	III-4	1.16	0.00	0.00
AL -4	New	0.08	23.11	3603.42	III-6	3.46	1.15	0.89
AL -5	New	0.06	24.70	4273.52	III-6	3.78	5.31	1.45
AL-6	New	0.78	15.56	3476.65	III-5	6.37	0.66	0.02
AL-7	New	0.53	26.09	3860.27	III-6	3.57	1.74	0.08
AL-8	Known	0.50	28.84	3318.25	III-5	4.75	0.00	0.06
AL-9	New	0.18	31.22	3870.47	III-6	2.02	0.10	0.00
AL-10	New	0.32	34.07	3793.84	III-6	0.00	0.05	0.00
AL-11	Known	0.76	22.42	3381.68	III-5	5.54	0.00	0.00
AL-12	Known	0.08	28.12	3789.90	III-6	0.86	0.00	0.06
AL-13	Known	0.43	30.40	3275.93	III-5	4.75	0.00	0.08
AL-14	New	0.02	15.77	3774.90	III-6	1.01	0.00	0.05
AL-15	New	0.45	26.38	4431.45	III-6	0.60	1.01	0.86
AL-16	New	0.71	24.07	4533.01	III-5	1.18	1.50	1.28
AL-17	New	0.14	28.41	4035.75	III-6	6.40	0.00	0.45
AL-18	New	0.06	21.61	3686.16	III-6	0.02	0.29	0.19
AL-19	New	0.49	25.89	4032.19	III-5	3.44	0.20	0.22
AL-20	New	0.86	23.35	3818.58	III-6	0.79	0.03	0.00
AL-21	New	0.20	35.22	3598.85	III-6	0.36	0.00	0.00
AL-22	New	0.26	26.43	3787.55	III-5	6.59	0.59	1.58
AL-23	New	0.24	28.89	3486.76	III-6	4.90	0.27	0.03
AL-24	Known	0.04	15.08	3797.88	III-5	4.38	0.68	0.45
AL-25	Known	0.22	21.17	3265.26	III-5	5.01	0.02	0.14

Supplementary Table S2 Correlation of active landslide deformation with various triggering factors. The correlation values are calculated from the Pearson correlation coefficients and pass the significance level of 5%. The correlation with earthquakes is the correlation coefficient between deformation velocity and cumulative PGA. The correlation with rainfall is the correlation coefficient between deformation velocity and cumulative rainfall. The correlation with human activity is the correlation coefficient between the area proportion of significant deformation and distance to roads. “null” means the correlation coefficient does not pass the significance test.

Landslide	Correlation with earthquakes	Correlation with rainfall	Correlation with human activity
AL-1	0.303	0.583	null
AL-2	null	0.558	-0.986
AL-3	0.724	0.493	null
AL -4	null	0.4	null
AL -5	null	0.584	null
AL-6	0.615	null	-0.829
AL-7	0.315	0.348	null
AL-8	null	0.748	null
AL-9	null	0.771	null
AL-10	null	0.612	null
AL-11	0.62	0.445	null
AL-12	null	0.577	-0.936
AL-13	0.621	null	null
AL-14	null	0.714	null
AL-15	null	0.574	null
AL-16	null	0.641	null
AL-17	null	0.398	null
AL-18	null	0.494	null
AL-19	null	0.506	null
AL-20	0.733	0.533	null
AL-21	null	0.577	-0.995
AL-22	null	0.683	-0.886
AL-23	null	0.626	-0.943
AL-24	null	0.699	null
AL-25	0.699	0.508	-0.895

Two landslides induced by different factors are selected as examples. AL-1 landslide (Figure S2) is situated on the southern bank of the Maiqu river with a distance of 240 m to the river, featuring the weak rock group of mudstone and shale and very steep terrain. The average slope angle of the significant deformation region reaches 41.81°. Mudstone and shale belong to clay rocks that are apt to be softened by water (FGB, 2020). Thus, under the combined function of rainwater infiltration and softening and river down-cutting and erosion, the effective stress and shear strength decreased, and the landslide became slowly moving along the weak sliding surface.

Moreover, earthquakes led to the relaxation and destruction of soil and rock mass and also contributed to the landslide development. As shown in Figure S2, the deformation velocity exhibits a high consistency with the rainfall variation, obviously increasing in the rainy season. During the dry season, the deformation was enhanced due to the relaxation of soil and rock mass caused by earthquakes.

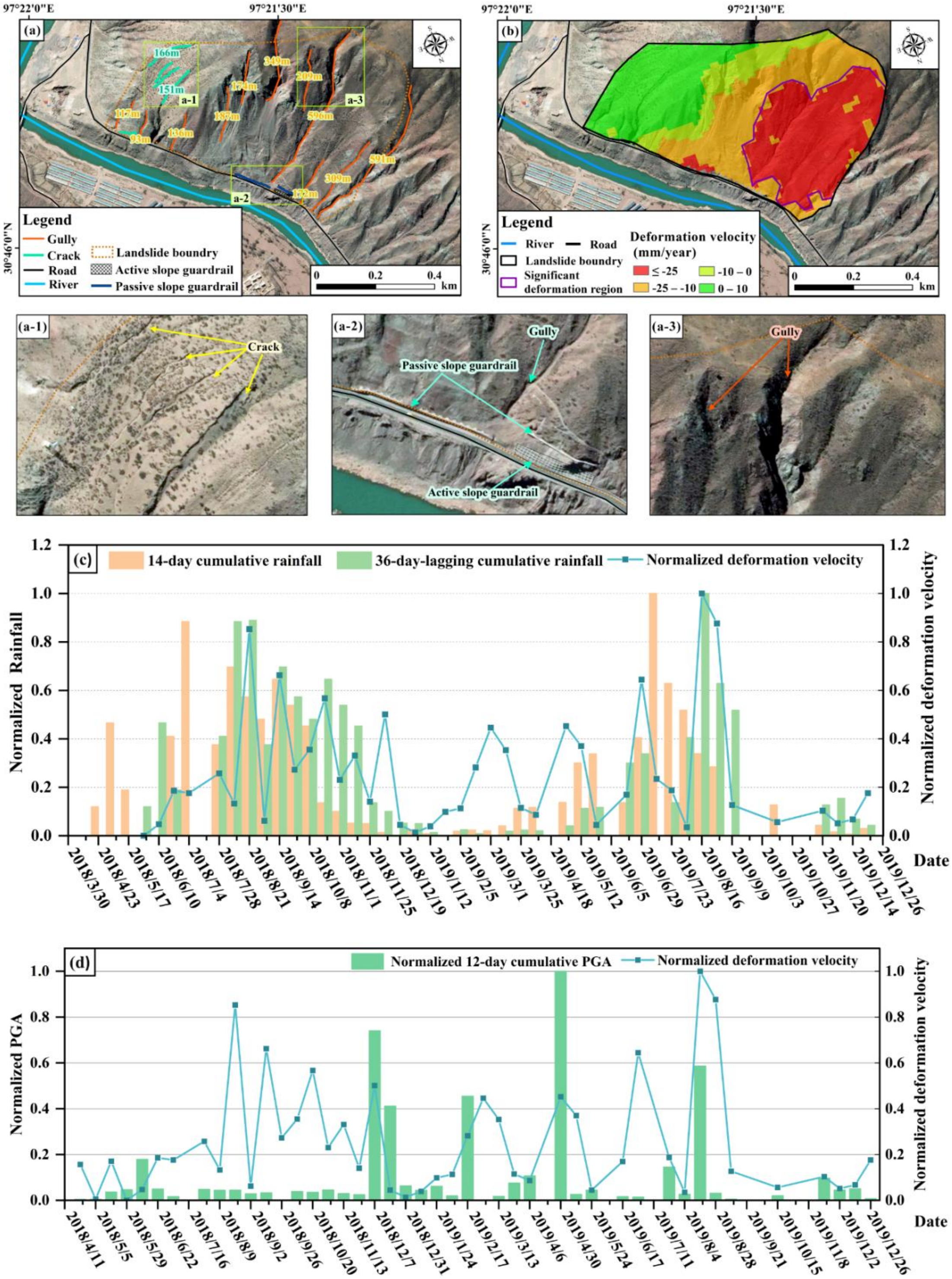

Supplementary Figure S2 Deformation characteristics and triggering factors of AL-1 Landslide. (a) Macroscopical deformation signs. (b) Deformation velocity monitored by the SBAS-InSAR

technique. (c) Relation between landslide deformation and rainfall. (d) Relationship between landslide deformation and earthquakes. The base images are three-dimensional Mapbox images with the resolution of 0.5 1m.

AL-21 landslide (Figure S3), a newly-discovered landslide, is developed in the rock assemblage of relatively hard quartz sandstone, siltstone, and volcanics. This type of rock belongs to brittle rocks and is liable to be weathered, featuring developed cleavages and cracks (FGB, 2020). Thus, rainwater washed the slope body and caused the densely-distributed gullies with the maximum width of ~4 m and the maximum length of ~252 m. Moreover, rainwater infiltrated through the cracks, concentrated on the aquiclude, and played a lubrication role (Hafizi et al., 2010). The endosmic rainwater saturated the soil and rock mass and increased their gravity and sliding force (Finlay et al., 1997; Wang, 2001; Yao et al., 2002). Furthermore, a road network was built on the slope body, and the slope excavation and explosion loosened and unloaded the soil and rock mass and promoted slope movement. Therefore, the active landslide was induced by the coupling action of precipitation and human engineering activity.

Moreover, the macroscopical deformation signs of two newly-discovered potential landslides are shown in Figure S4.

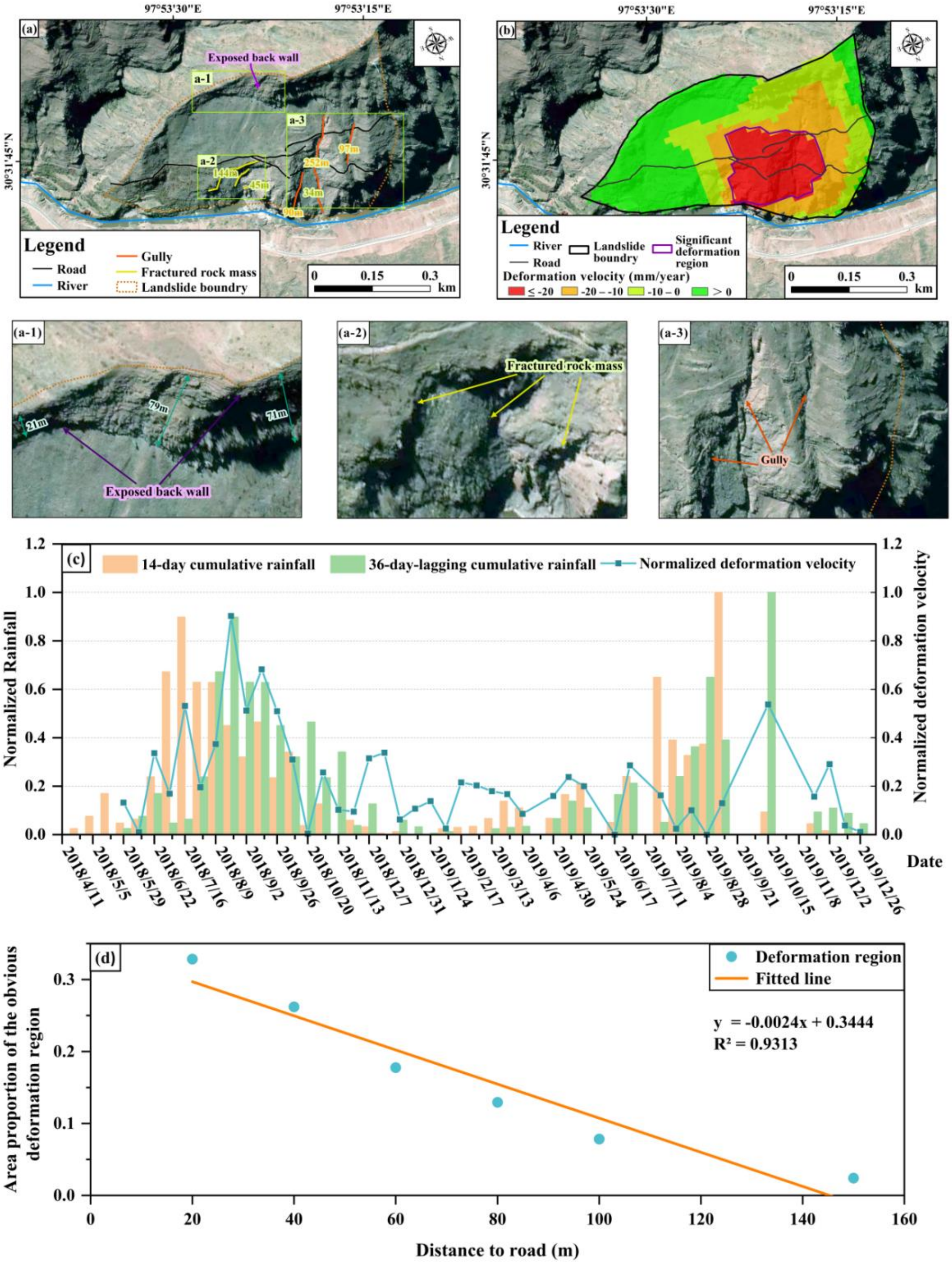

Supplementary Figure S3 Deformation monitoring and inducing factors of AL-21 landslide. (a) Steep slope, developed cracks and gullies, and fractured rock mass. (b) Deformation velocity observed by InSAR. (c) Link between landslide deformation and precipitation. (d) Relation between landslide deformation and road construction. The base images are three-dimensional Mapbox images with the resolution of 0.51m.

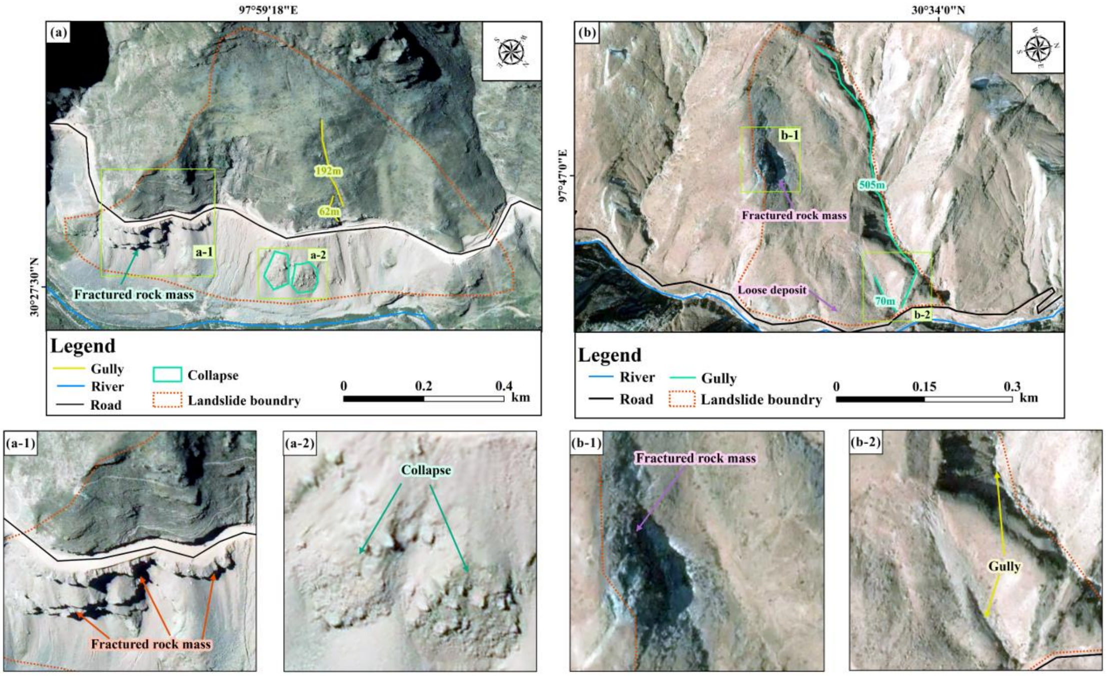

Supplementary Figure S4 Macroscopical deformation signs of two potential landslide examples (AL-10 and AL-20). The base images are three-dimensional Mapbox images with the resolution of 0.5 1m.

Online Resource 3: Validation of the identified active landslides

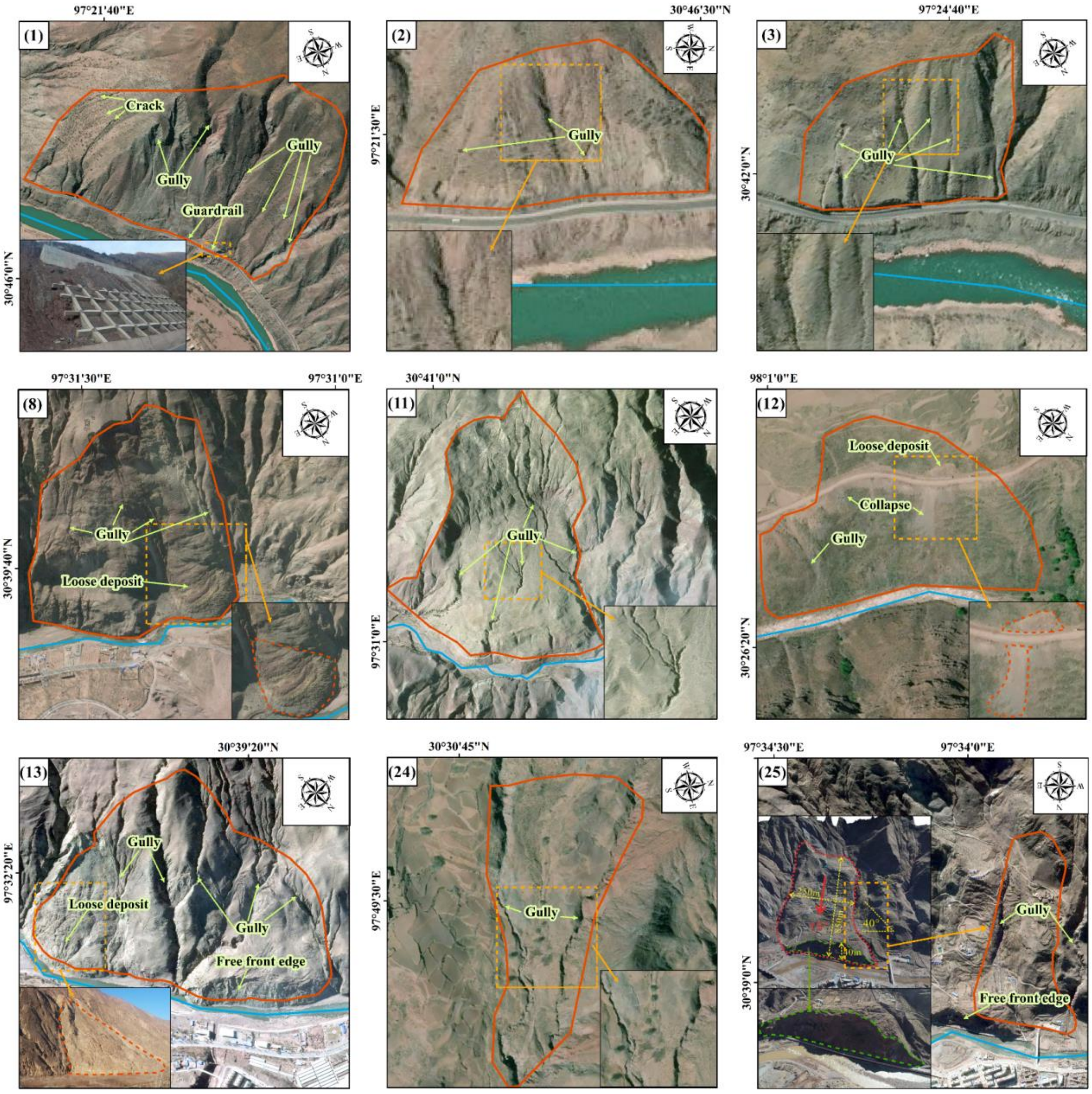

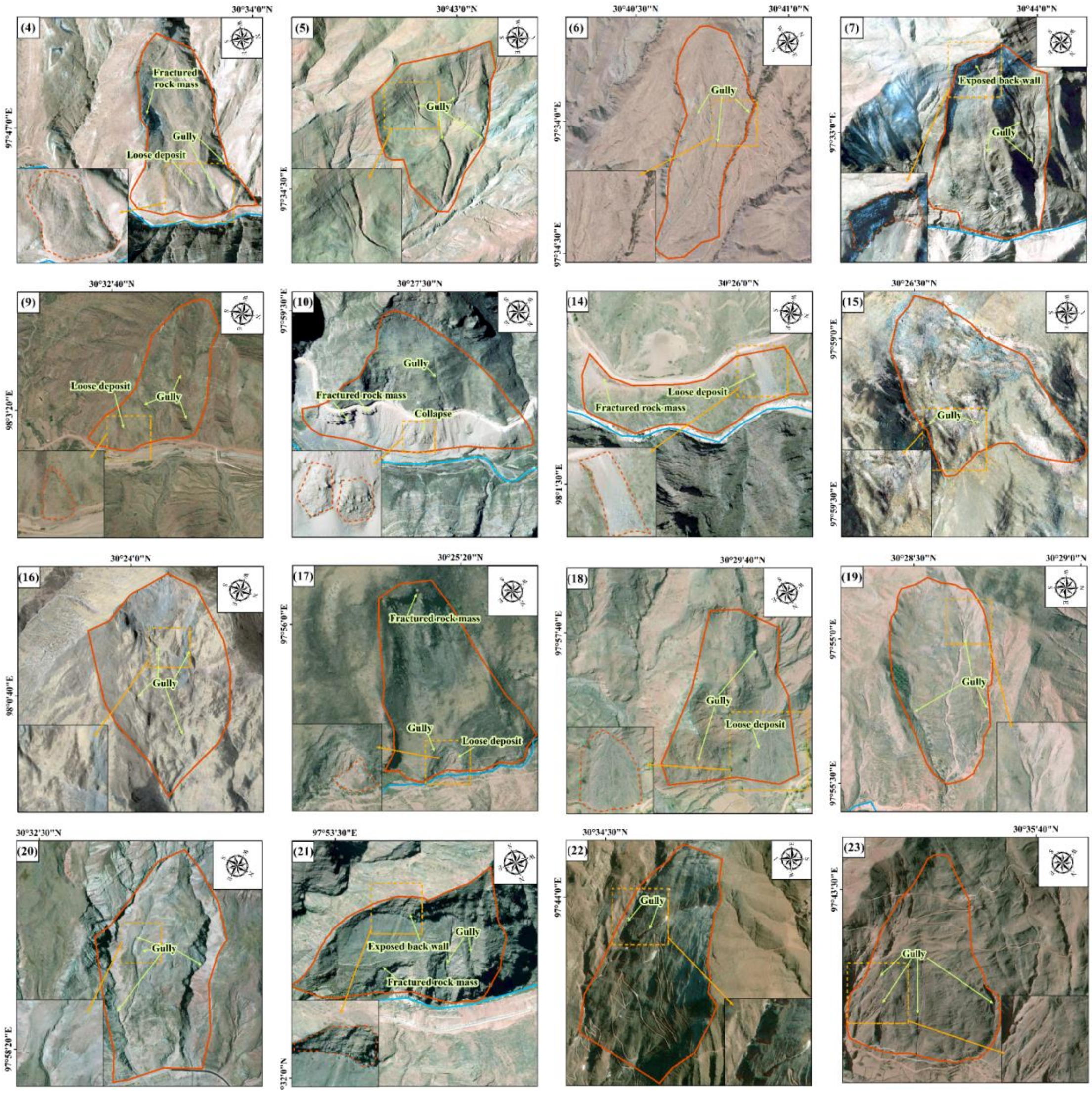

Supplementary Figure S5 Validation of 25 identified active landslides via field survey (FGB, 2020), UAV images (FGB, 2020), and 3D Mapbox images. The field survey photos and UAV images are provided by Bureau of Geology and Mineral Exploration and Development of Tibet Autonomous Region.

Online Resource 4: Advancement and rationality of the suggested criteria for potential landslide identification

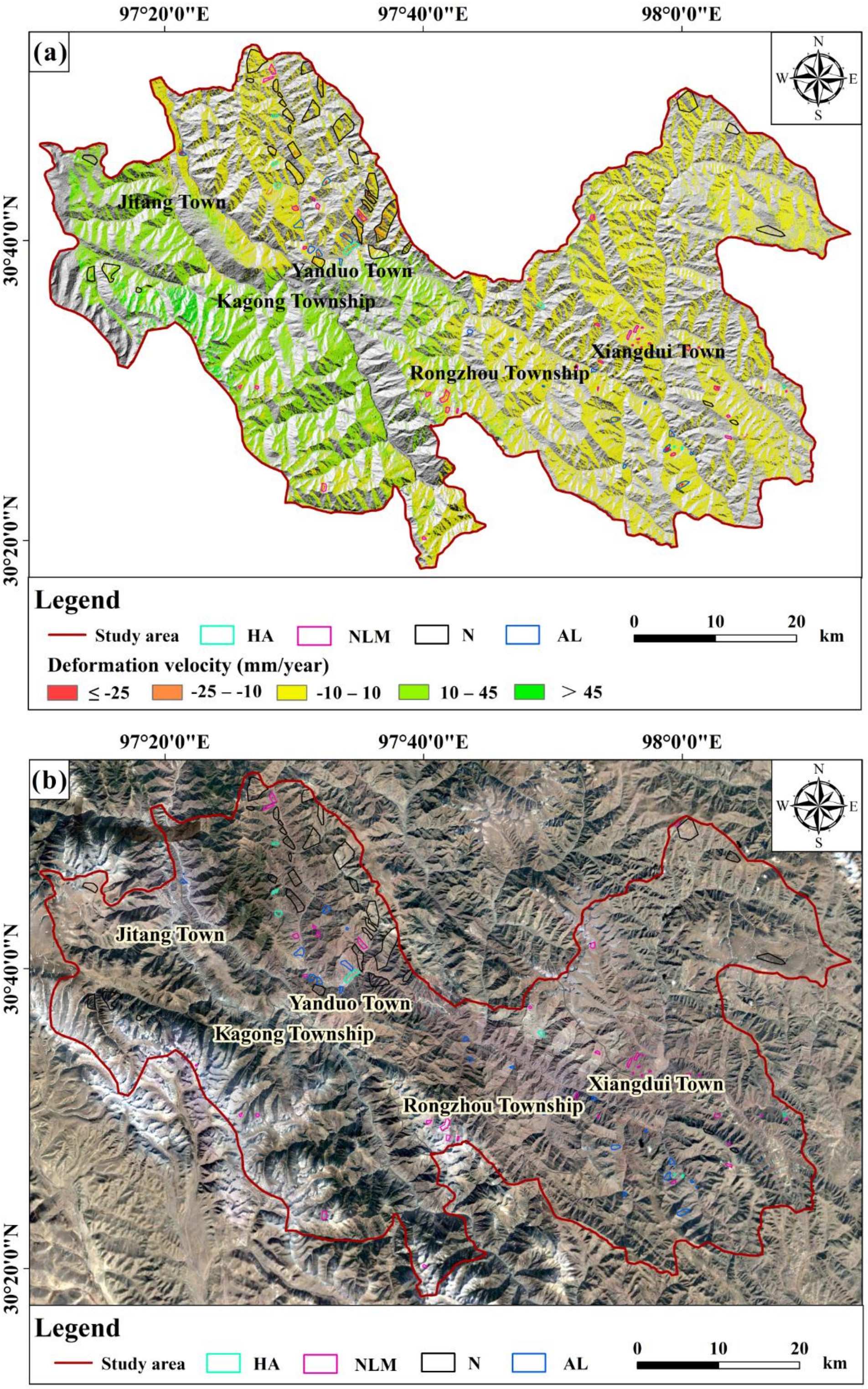

Supplementary Figure S6 False alarm regions generated by SBAS-InSAR technique superimposed on (a) Deformation velocity map and (b) Google Earth images shot on February 2, 2015; March

16, 2015; November 7, 2020; and November 11, 2020, respectively. HA means the false alarm areas associated with human engineering activity, NLM indicates the false alarm areas without landslide geomorphological characteristics, N represents the false alarm regions from noises, and AL indicates active landslides.

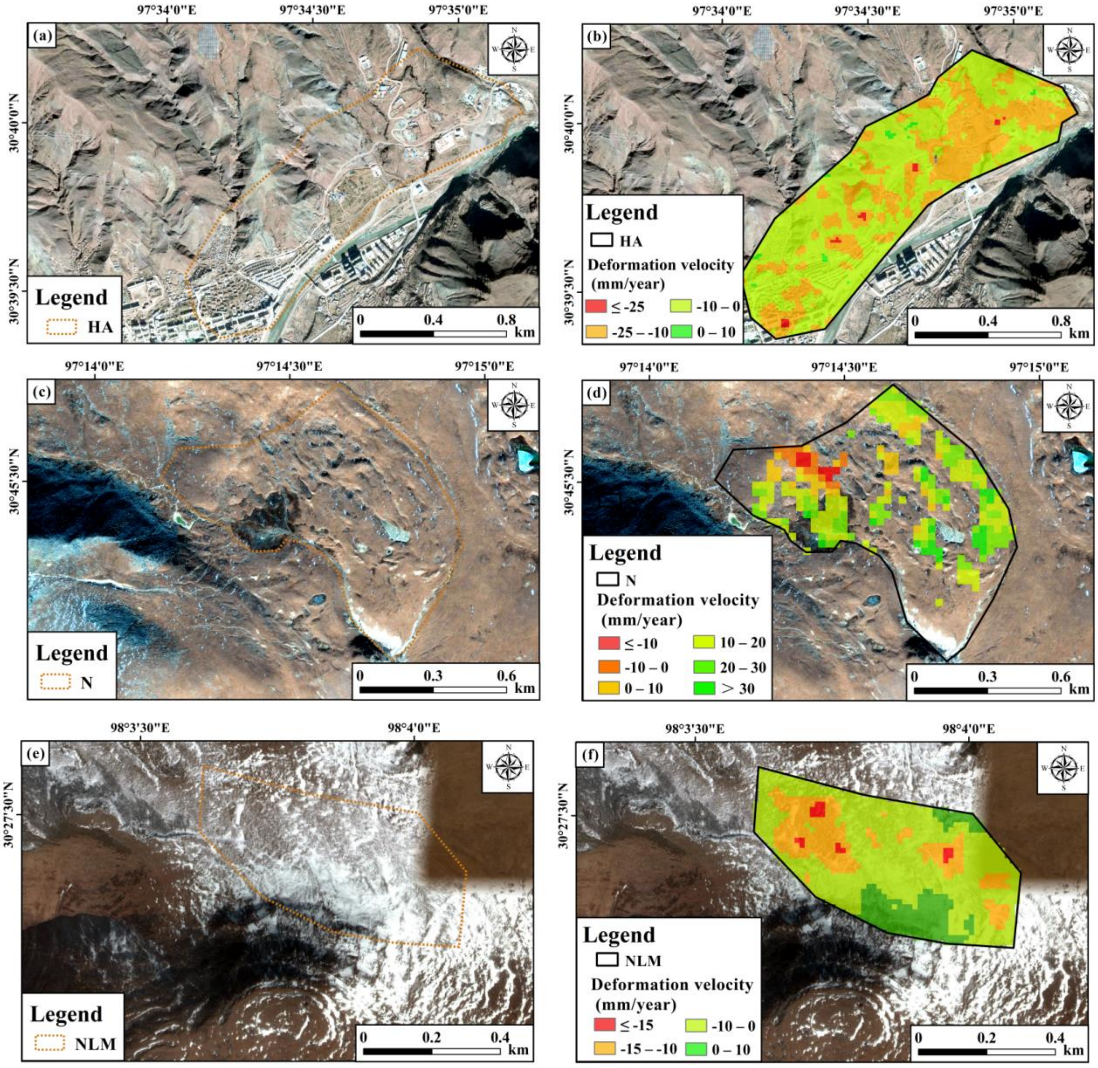

Supplementary Figure S7 Three examples of false alarms. See Figure S6 for the meaning of the legends. The base images are Mapbox images.

Online Resource 4: Collinearity analysis among the initial indices of landslide susceptibility evaluation

Supplementary Table S3 Collinearity relation among various disaster-controlling and disaster-triggering factors. The abbreviations include: EVC=Elevation variation coefficient; TWI=Topographic wetness index; and NDVI=Normalized difference vegetation index.

No.	Influencing factor	VIF	TOL
1	Elevation	0.357	2.798
2	Slope angle	0.132	7.568
3	Slope aspect	0.812	1.232
4	Curvature	0.849	1.178
5	Surface roughness	0.195	5.137
6	Surface cutting depth	0.155	6.452
7	Relief amplitude	0.183	5.465
8	EVC	0.164	6.087
9	Stratum	0.857	1.167
10	Distance to fault	0.806	1.240
11	Distance to river	0.216	4.625
12	TWI	0.602	1.662
13	Cumulative rainfall	0.461	2.170
14	Seismic kernel density	0.606	1.649
15	Distance to road	0.205	4.871
16	NDVI	0.748	1.337
17	Land use	0.803	1.246

References

Finlay, P. J., Fell, R., Maguire, P. K., 1997. The relationship between the probability of landslide occurrence and rainfall. Canadian Geotechnical Journal, 34(6): 811–824.

Hafizi, M. K., Abbassi, B., Ashtari, T. A., 2010. Safety assessment of landslides by electricaltomography: A case study from Ardabil, northwestern Iran. Journal of the Earth and Space Physics, 36(1): 935.

Wang, F., 2001. Mechanism of rapid landslides：excess pore pressure generation caused by grain crushing. Journal of Changchun University of Science and Technology, 31(1): 64–69 (in Chinese with English Abstract).

Yao, H. L., Zheng, S. H., Li, W. B., et al., 2002. Parametric study on the effect of rain infiltration on stability of unsaturated expansive soil slope. Chinese Journal of Rock Mechanics and Engineering, 21(7): 1034–1039 (in Chinese with English Abstract).
